# Global trends in the application of fluorescence imaging in pancreatic diseases: a bibliometric and knowledge graph analysis

**DOI:** 10.3389/fonc.2024.1383798

**Published:** 2024-07-19

**Authors:** Quanneng Luo, Xiong Teng, ManXiong Dai, Jun Yang, Wei Cheng, Kang Chen, Lei Zhou

**Affiliations:** ^1^ Department of Hepatobiliary Surgery, Hunan Provincial People’s Hospital (The First Affiliated Hospital of Hunan Normal University), Changsha, Hunan, China; ^2^ Hunan Schistosomiasis Control Center (Hunan Third People’s Hospital), Yueyang, Hunan, China

**Keywords:** pancreatic diseases, fluorescence imaging technology, bibliometric, ICG, trend

## Abstract

**Background:**

In recent years, with the continuous development of fluorescence imaging technology, research on its application in pancreatic diseases has surged. This area is currently of high research interest and holds the potential to become a non-invasive and effective tool in the diagnosis and treatment of pancreatic diseases. The objective of this study is to explore the hotspots and trends in the field of fluorescence imaging technology applications in pancreatic diseases from 2003 to 2023 through bibliometric and visual analysis.

**Methods:**

This study utilized the Web of Science (core collection) to identify publications related to the application of fluorescence imaging technology in pancreatic diseases from 2003 to 2023. Tools such as CiteSpace (V 6.2.R6), VOSviewer (v1.6.20), and R Studio (Bibliometrix: R-tool version 4.1.4) were employed to analyze various dimensions including publication count, countries, institutions, journals, authors, co-cited references, keywords, burst words, and references.

**Results:**

A comprehensive analysis was conducted on 913 papers published from January 1, 2003, to December 1, 2023, on the application of fluorescence imaging technology in pancreatic diseases. The number of publications in this field has rapidly increased, with the United States being the central hub. The University of California, San Diego emerged as the most active institution. “Biomaterials” was identified as the most influential journal. Authors with the most publications and the highest average citations per article are Hoffman, Robert M. and Luiken, George A., respectively. Keywords such as pancreatic cancer, cancer, expression, indocyanine green, and nanoparticles received widespread attention, with indocyanine green and nanoparticles being current active research hotspots in the field.

**Conclusion:**

This study is the first bibliometric analysis in the field of fluorescence imaging technology applications in pancreatic diseases. Our data will facilitate a better understanding of the developmental trends, identification of research hotspots, and direction in this field. The findings provide practical information for other scholars to grasp key directions and cutting-edge insights.

## 1 Introduction

In recent years, fluorescence imaging technology’s application in clinical practice has grown, capable of obtaining a comprehensive spectrum of imaging information concerning tissues at the morphological, functional, and molecular levels simultaneously. Using optical imaging techniques, it is possible to delineate specific structural, functional, metabolic, and molecular details pertaining to cells and tissues, including hemoglobin concentration, oxygen saturation, blood flow, vessel density, and enzyme activity (1). Fluorescence imaging has the advantages of high sensitivity, real-time imaging, convenience, and absence of ionizing radiation (2, 3). Near-infrared two-region fluorescence imaging (NIR-II, 1000-1700 nm) mitigates the issues of significant tissue absorption, scattering, and autofluorescence inherent in traditional fluorescence (NIR-I, 400-900 nm), thereby offering greater tissue penetration depth and enhanced spatial resolution in *in vivo* imaging (4). Applications of fluorescence imaging extend to various diseases, such as those affecting the hepatobiliary system, gastrointestinal tract, mammary glands, kidneys, and bones. As a retroperitoneal organ, the pancreas presents lesions that are typically concealed, rendering early-stage diagnosis challenging. Traditional medical imaging modalities, such as CT and MRI, are often inadequate for the early and real-time detection of conditions, particularly pancreatic cancer, which is highly malignant with an increasing incidence rate and offers a poor prognosis. Globally, pancreatic cancer is the third leading cause of cancer-related mortality, with a 5-year survival rate of a mere 12%, and an average survival time of less than 6 months (5). With the ongoing advancements in fluorescence imaging technology, research into its application for pancreatic diseases has substantially increased, currently establishing itself as an area of intense research interest with the potential to evolve into a noninvasive and effective tool for the diagnosis and management of pancreatic disease.

Numerous approaches exist for the systematic review of research fields, and one particularly prevalent approach is bibliometric analysis, which employs quantitative research methodologies based on literature publications, citations, and textual data to describe and analyze the dynamics and advancements within specific disciplines or research fields (6). Bibliometric studies yield not only descriptive statistics but also undertake network analyses of keywords, texts, citations, authors, institutions, and their interconnections. These analyses examine the frequency, correlations, centrality, and clustering of authorship and textual data, thereby often facilitating scholarly exploration of evolutionary patterns, publication trends, and authorship networks within specific topics or theses. Furthermore, these analyses enable the identification of research priorities and emerging themes within the field.

Existing bibliometric analyses offer insights into numerous medical research areas, including oncology (7), orthopedics (8), respiratory diseases (9), cardiovascular medicine (10), neurosurgery (11), medical imaging (12), pediatrics (13), and gastroenterology (14), among others. To date, the literature lacks bibliometric analysis focusing on the application of fluorescence imaging specifically in pancreatic studies. To comprehensively understand the dynamics and hotspots of fluorescence imaging technology’s application in pancreatic studies, and to offer valuable references for researchers in this area, the author has conducted a bibliometric summary of significant academic literature over the past two decades, analyzing and outlining the development trends and hotspot distribution within this field, and suggesting ideas for future reforms and advancements.

## 2 Methodologies

### 2.1 Search strategy

**Figure 1:**
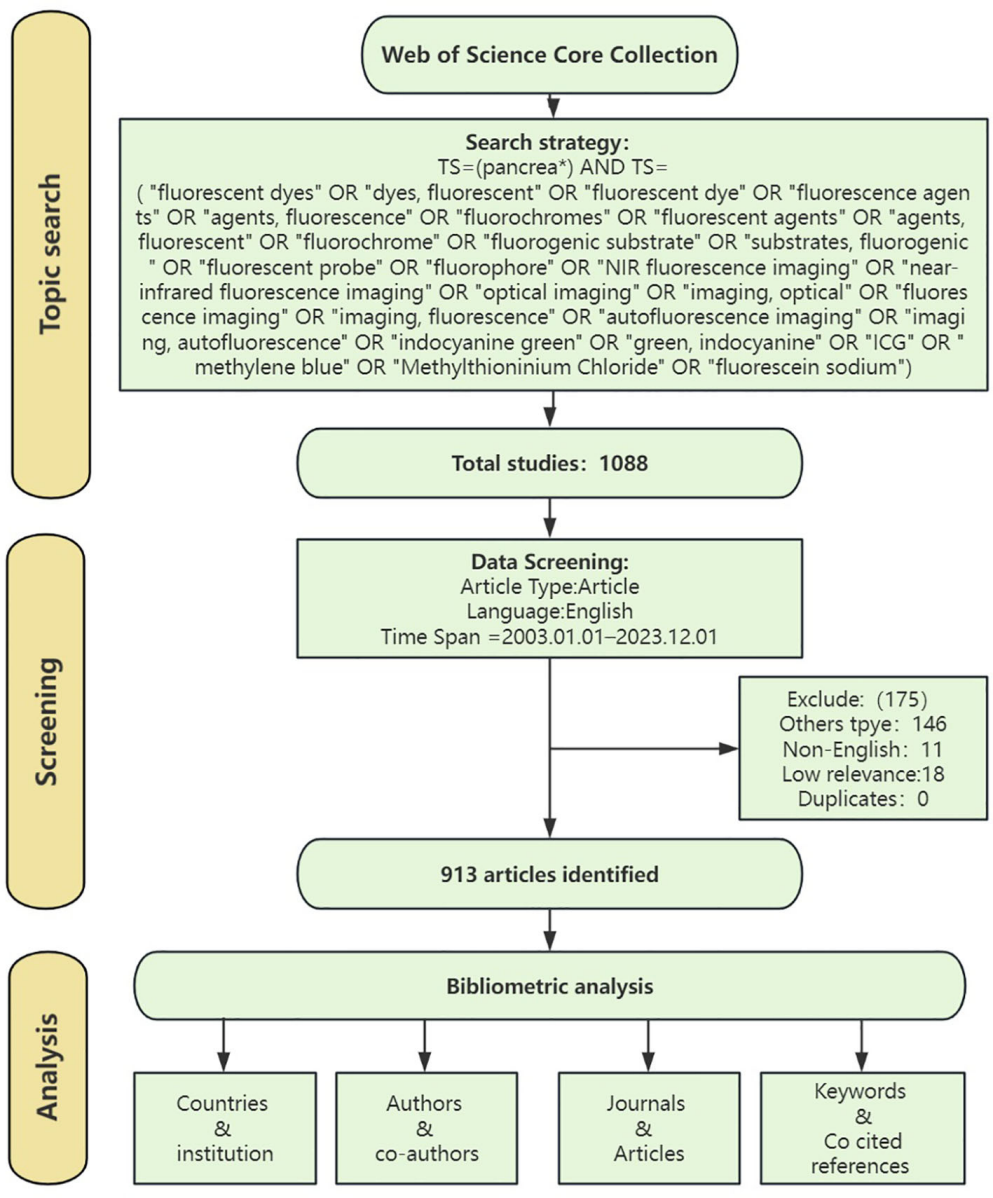
Screening flowchart.

Web of Science (core collection) was selected as the data source for this paper, which has been recognized by many researchers as a high-quality digital literature resource database. In order to ensure the comprehensiveness and accuracy of the retrieved data, and to avoid the errors caused by database update, this paper was searched on December 01, 2023, to retrieve the research literature about the application of fluorescent imaging technology in the field of pancreas from the core database of Web of Science, and the searching strategy was as follows: TS=(pancrea*) AND TS=(“fluorescent dyes “ OR “dyes, fluorescent” OR “fluorescent dye” OR “fluorescence agents” OR “agents, fluorescence” OR “fluorochromes” OR “fluorescent agents” OR “ agents, fluorescent” OR “fluorochrome” OR “fluorogenic substrate” OR “substrates, fluorogenic” OR “fluorescent probe” OR “fluorophore” OR “NIR fluorescence imaging” OR “near-infrared fluorescence imaging” OR “optical imaging” OR “imaging, optical” OR “fluorescence imaging” OR “imaging, fluorescence” OR “autofluorescence imaging” OR “imaging, autofluorescence” OR “indocyanine green” OR “green, indocyanine” OR “ICG” OR “methylene blue” OR “Methylthionine blue” OR “Methylthioninium Chloride” OR “fluorescein sodium”), a total of 1,088 related documents were searched, with the limiting language set to English, the document type set to Article, and the time span from January 01, 2003 to December 01, 2023 After limiting the scope, a total of 931 relevant documents were found. The two researchers screened the search results again, and 913 articles remained after removing the less relevant and duplicated articles, and the process of literature search is shown in **Figure 1**.

### 2.2 Data extraction and analysis

The data were downloaded and exported from Wos (core collection) in the formats of “Plain text file”, “BibTex”, and “Tab delimited file”, and the data were selected as Full Record and Cited References, and then imported into CiteSpace (V 6.2.R6), VOSviewer (v1.6.20), and R Studio (Bibliometrix: R-tool version 4.1.4) for quantitative evaluation.

### 2.3 Introduction to the tool

CiteSpace, a JAVA-based scientometrics knowledge mapping tool for academic research, was developed by Professor Chao-Mei Chen in the United States. It is used to visualize and analyze scientific literature in the fields of bibliometrics and citation analysis and is widely employed across various research fields in the natural and social sciences. Researchers commonly use CiteSpace to gain insight into the structure of scholarly literature, identify influential works and authors, and track the development of research areas over time. This tool is frequently employed in the fields of bibliometrics, scientometrics, and information visualization (15).

VOSviewer utilizes a probabilistic-based data normalization method and offers various visualization views in the fields of keywords, co-institutions, and co-authors, including Network Visualization, Overlay Visualization, and Density Visualization, noted for its easy mapping and beautiful images (16).

R Studio, an efficient and integrated version of the R language, is used for data analysis and processing, graphical plotting, and reporting. In this study, R Studio is utilized for visualization and analysis.

Each of these software programs possesses its own unique strengths and can effectively complement the others. The above three tools were employed to analyze data using descriptive statistics. The analytical indices included the number of published articles, year of publication, country of publication, publishing institution, journal, author, keywords, references, citation bursts, total citations, average citations, h-index, among others.

## 3 Results

The 913 papers used in this study came from 5772 authors from 1200 institutions in 52 countries, published in 417 journals, and cited 31052 citations from 4089 journals.

### 3.1 Country-based analysis of publications

**Figure 2:**
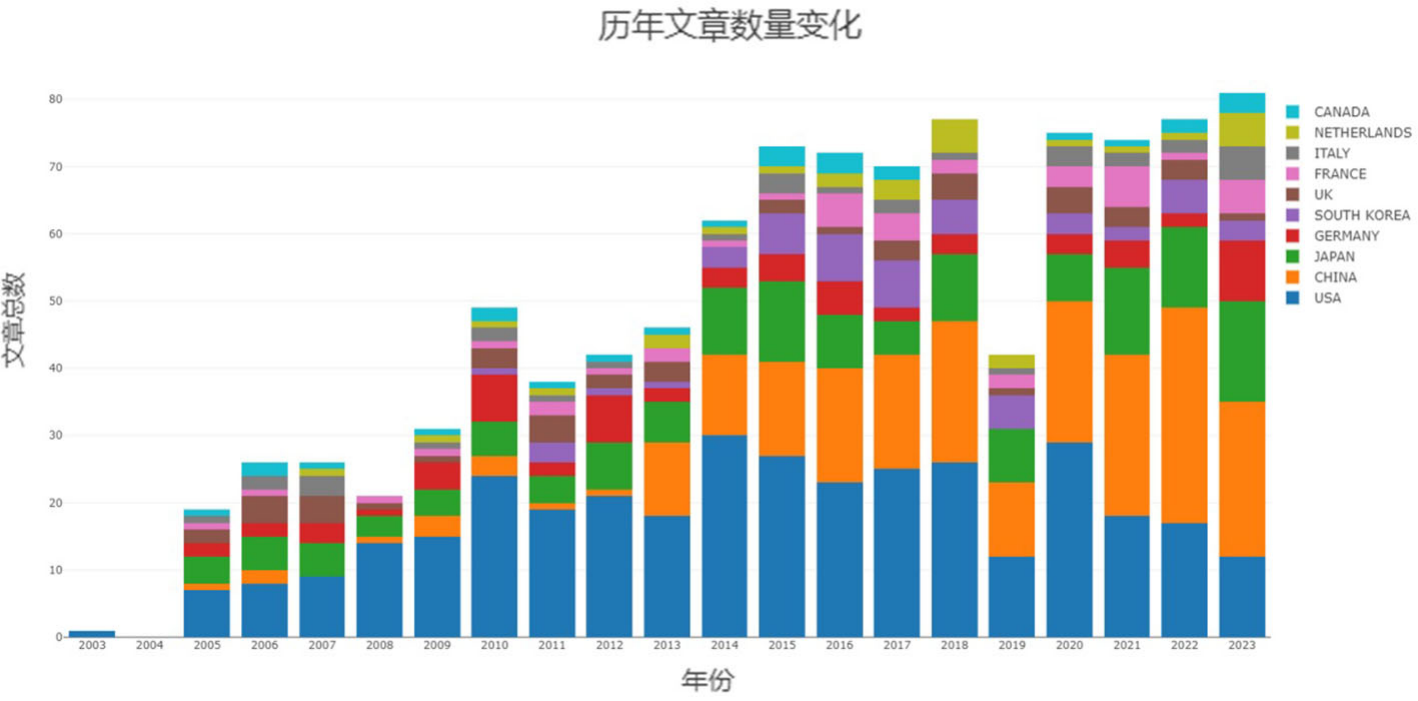
Year-on-year growth in the number of publications in the top 10 countries.

Author origins were statistically analyzed, and **Figure 2** illustrates the temporal distribution of publications in the field of pancreatic fluorescence imaging technology. Notably, there has been an increasing trend in publications, especially since 2014, indicating growing scholarly interest in this research area. A majority of these articles were published in the United States, China, and Japan.

**Figure 3:**
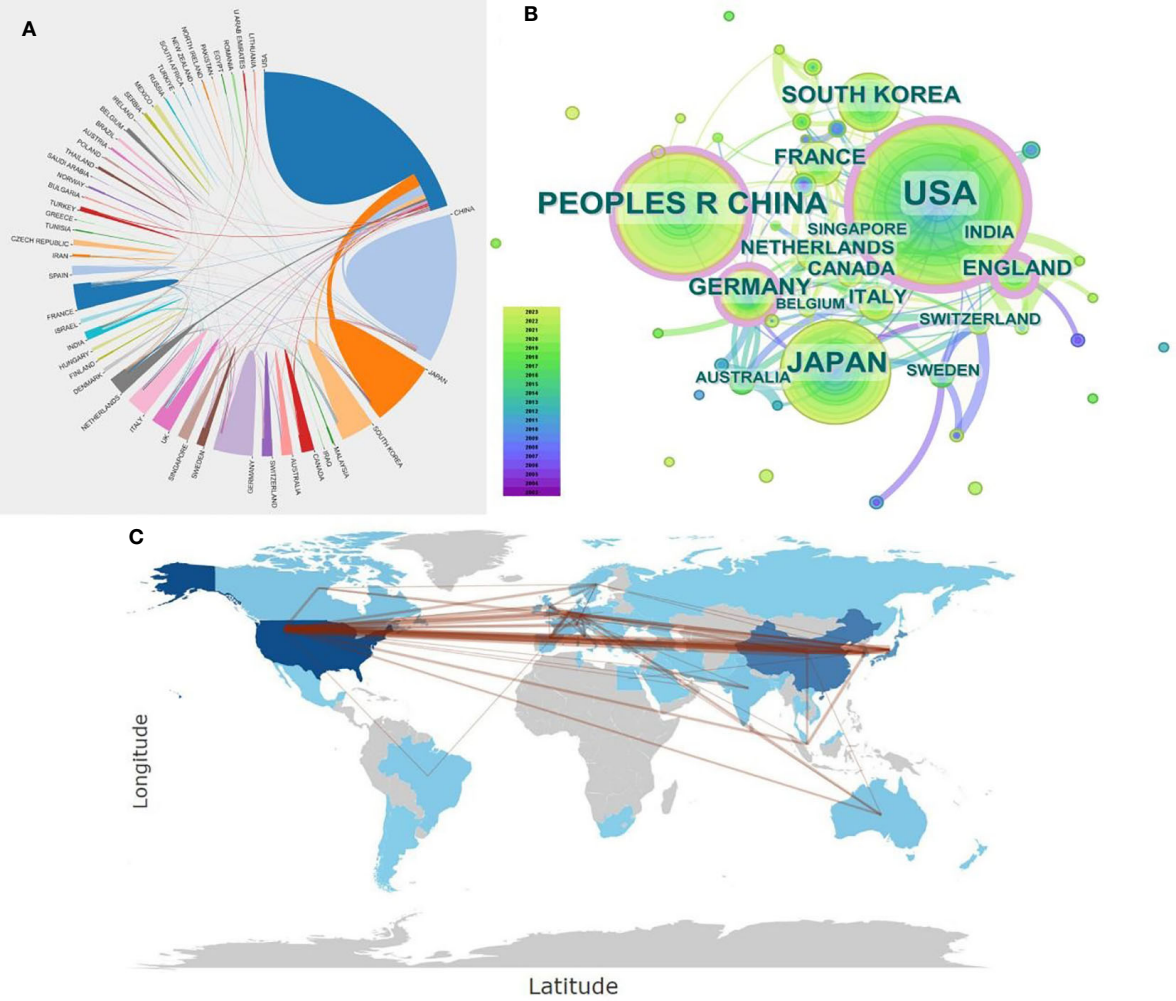
**(A)** National Document Cooperation Relationship Diagram: Utilizing Bibliometrix: R-tool version 4.1.4 to visualize the publication collaboration relationships among countries, where the thickness of the lines denotes the strength of collaboration between countries. **(B)** Country Cooperation Network Co-occurrence Graph: Employing CiteSpace to visualize a co-occurrence network of the top 10% of countries in terms of publication volume. Each node represents a country, with the size of the node indicating the volume of publications. The different widths of tree rings on a node represent articles published in different years, while the color of the lines between nodes indicates the year of collaborative publications between institutions. The thickness signifies the frequency of collaboration. A purple outer ring signals centrality, with a thicker purple outer ring denoting a more significant role of the country in the network map. **(C)** Country Cooperation Map: Using Bibliometrix: R-tool version 4.1.4 to visualize the publication collaboration relationships among countries. The darker the color, the higher the volume of publications, and lines indicate the strength of collaboration between countries.

To ascertain which countries have made the most significant contributions to this research field, the present study analyzed publication data from 52 countries. Initially, CiteSpace was employed to visualize the top 10% of countries by publication count. The resulting network, depicted in **Figure 3B**, comprises 49 nodes and 119 connecting lines. Larger nodes signify a greater number of publications. Additionally, the nodes’ annual rings’ varying widths denote the number of articles published in different years, while the lines’ colors and thicknesses correspond to the years of collaborative publications and the volume of collaboration, respectively. A purple outer circle indicates a centrality degree greater than 0.1, with its thickness reflecting the country’s prominence within this research field. The figure reveals a highly uneven distribution among contributing countries, with a pronounced ‘top effect’ indicating dominance by a select few. A few countries, particularly the United States, China, and Japan, contribute the majority of scholarly articles, with the top two countries alone accounting for over 60% of total publications. The purple circle highlights the United States’ preeminent centrality and influence. Moreover, the United Kingdom, China, and Germany also exhibit centralities above 0.1. **Figures 3A, C** illustrates relatively strong collaborative ties among these nations, among which the United States is the most closely connected with the other countries.

Further analysis was conducted on the most productive countries in this field, as presented in **Table 1**, which lists the top 10 countries by publication count. Analysis of the data reveals that scholars from the United States have contributed the highest number of research papers in this field, totaling 344 papers. This accounts for 37.7% of the field’s publications, with an impressive citation count of 12,851. The United States also leads in the average number of citations per article, at 37.36. China ranks second with 212 articles, though it has a comparatively lower citation rate. Citation count is widely regarded as an indicator of an article’s impact and quality; thus, to enhance its influence in this field, China needs to improve the quality of its research publications.

**Table 1:** Top 10 countries in terms of number of publications.

Country	Articles	Citation	Average Article Citations	Centrality
USA	344	12851	37.36	0.33
CHINA	212	4549	21.46	0.18
JAPAN	141	3424	24.28	0.09
GERMANY	64	1184	18.50	0.12
SOUTH KOREA	51	1014	19.88	0.04
UNITED KINGDOM	46	1382	30.04	0.25
FRANCE	38	962	25.32	0.06
ITALY	32	717	22.41	0.06
NETHERLANDS	28	999	35.68	0.07
CANADA	25	538	21.52	0.01

Subsequently, we analyzed the publication trends of the top 10 countries in this field. The analysis indicates a year-over-year upward trend in research for each country, particularly in the United States, China, and Japan, which account for the largest share of annual publications. Since 2013, China has progressively gained momentum in the field, eventually matching the United States in publication volume post-2020, as illustrated in **Figure 2**.

### 3.2 Agency-based analysis of publications

**Figure 4:**
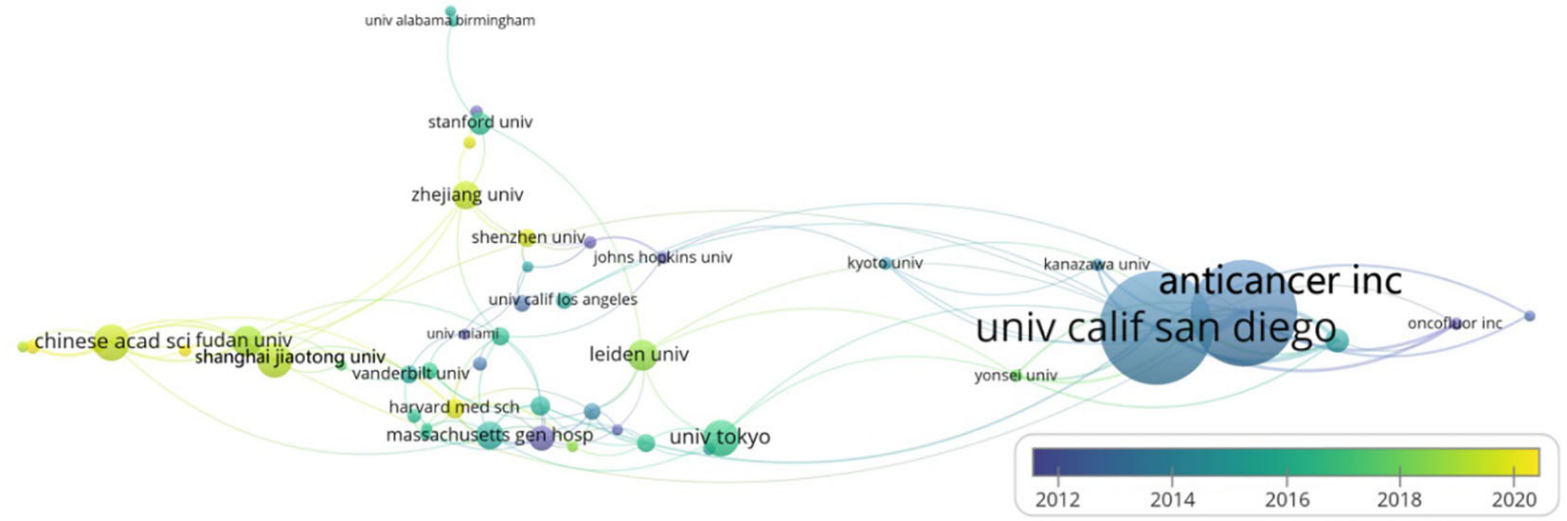
Institutional Cooperation Network Co-Occurrence Graph: VOSviewer is employed to visualize the co-occurrence network of institutions with a publication count of six or more. Each node symbolizes an institution, where the node’s size reflects the number of articles, and the node’s varying colors denote the publication years of the articles. The connecting lines’ colors represent the years of collaborative publications between institutions, and their thickness signifies the frequency of cooperation.

Research institutions serve as pivotal drivers of research, with the perspectives of leading institutions often shaping the academic discourse in the field. As indicated in **Table 2**, the most prolific institution in this field is the University of California, San Diego, with 61 publications, followed by Anticancer Inc. (57 publications) and the Chinese Academy of Sciences (20 publications). In total, 53 institutions have published at least six articles each in this field. Among the top ten most active institutions, four are based in the United States and another four in China. Of these, Massachusetts General Hospital achieved the highest average citation rate, with 15 articles averaging 43.40 citations each, followed by Leiden University (17 articles, 41.65 citations each) and Harvard University (14 articles, 36.93 citations each). In terms of centrality, Harvard University leads with a centrality score of 0.29, followed by the Chinese Academy of Sciences (0.15) and Anticancer Inc. (0.10). Additionally, the data indicates that U.S. institutions primarily published their papers around 2010, whereas Chinese institutions have been more prolific in recent years, with a focus around 2020 (**Figure 4**).Cooperation between institutions is predominantly domestic, with limited transnational collaborations. Enhancing international academic exchanges could foster novel insights and advancements in this field.

**Table 2:** Top 10 institutions in terms of the number of articles issued.

Institution	Articles	Citations	Average Article Citations	Country	Centrality
Univ Calif San Diego	61	1961	32.15	USA	0.07
Anticancer Inc	57	1868	32.77	USA	0.10
Chinese Acad Sci	20	642	32.10	CHINA	0.15
Univ Tokyo	20	680	34.00	JAPAN	0.03
Shanghai Jiao Tong Univ	19	472	24.84	CHINA	0.02
Fudan Univ	17	351	20.65	CHINA	0.04
Leiden Univ	17	708	41.65	NETHERLANDS	0.00
Massachusetts Gen Hosp	15	651	43.40	USA	0.01
Zhejiang Univ	15	538	35.87	CHINA	0.02
Harvard Univ	14	517	36.93	USA	0.29

### 3.3 Author-based analysis of publications

Reviewing the works of influential authors in a field offers an expedited approach to comprehensively understanding its classic theories. The h-index, also referred to as the h-factor, represents a novel metric for assessing scholarly achievement. Devised by Hirsch, the h-index quantifies a researcher’s impact by indicating that a set number (h) of their publications have each received at least h citations. This index is considered a relatively precise indicator of an individual’s academic accomplishments. A higher h-index suggests greater influence and impact of a researcher’s publications.

**Figure 5:**
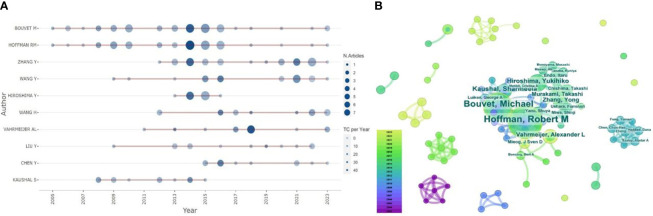
**(A)** Highly Productive Authors Posting Trends: Visualizing Highly Productive Authors Posting Trends with Bibliometrix: R-tool version 4.1.4. **(B)** Author Collaboration Network Co-occurrence Graph: Using CiteSpace to set the g-index (k=5), we visualize the co-occurrence network of authors, where each node symbolizes an author, with the node’s size reflecting the publication count, and the varying widths of the node’s annual rings denote the articles published across different years. The connecting lines’ color signifies the year of the authors’ collaborative publications, and their thickness indicates the collaboration frequency.

The top 10 most productive authors in this field include seven from the United States (**Table 3**), alongside one each from Japan, the Netherlands, and China, illustrating the significant influence of U.S. scholars in leading the field’s development. The most prolific author is Robert M. Hoffman (h-index(Wos)=76), an American scholar, with 53 papers published between January 2003 and December 2023, accumulating 1,696 citations and an average of 32.0 citations per paper. 2014 marked his peak year with over seven publications, significantly contributing to the advancement of research in the discipline (**Figure 5A**). Second in productivity is Michael Bouvet (h-index(Wos)=54), another American scholar, with 44 publications and 1,484 citations, averaging approximately 33.73 citations per article. Four of the seven U.S. scholars are affiliated with the University of California, San Diego, underscoring this institution’s prominent role as a leading publisher in the field. The scholar with the highest average citation per article is George A. Luiken (h-index(Wos)=12) from the United States, who has published seven articles, receiving a total of 509 citations, averaging 72.71 citations per article. Despite publishing fewer articles than the aforementioned scholars, Luiken’s work has garnered higher recognition within the field. The co-occurrence diagram reveals close collaborative relationships among these authors, evidenced by numerous co-authored articles (**Figure 5B**). This collaboration not only guides their scientific research but also optimizes their joint efforts, thereby fostering the discipline’s development and progress.

**Table 3:** Top 10 authors with the highest number of publications.

Author	Articles	Citation	Average Article Citations	Country	h-index(Wos)
Hoffman, Robert m.	53	1696	32.00	USA	76
Bouvet, Michael	44	1484	33.73	USA	54
Hiroshima, Yukihiko	14	426	30.43	Japan	32
Kaushal, Sharmeela	14	675	48.21	USA	24
Vahrmeijer, Alexander l.	12	615	51.25	NETHERLANDS	52
Zhang, Yong	12	357	29.75	USA	32
Murakami, Takashi	11	381	34.64	USA	33
Yong, Ken-Tye	8	529	66.13	CHINA	67
Choi, Hak Soo	7	429	61.29	USA	54
Luiken, George a.	7	509	72.71	USA	12

### 3.4 Analysis of periodical-based publications

**Figure 6:**
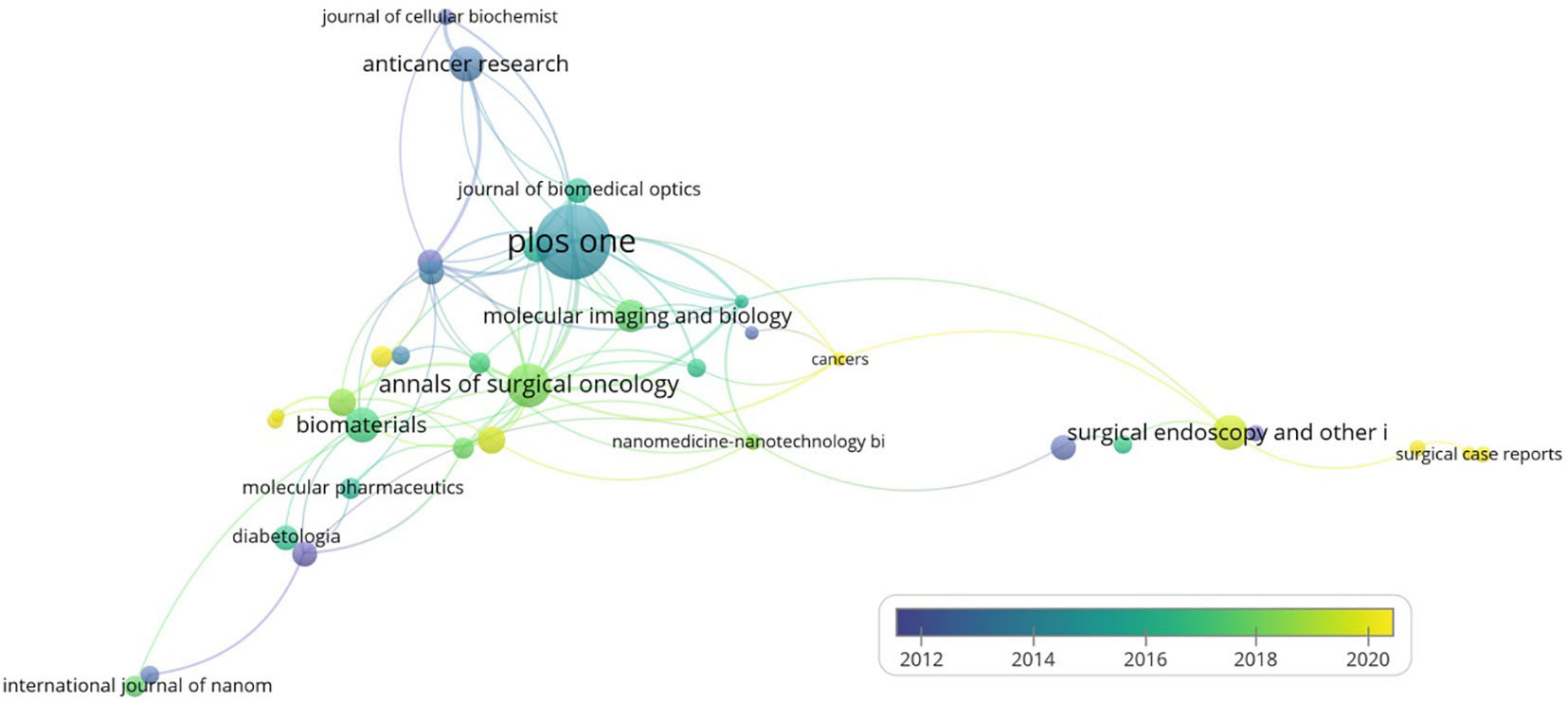
Journal collaboration network co-occurrence graph: co-occurrence network visualization of journals with more than 5 published articles using Vosviwer. Larger nodes indicate a higher volume of publications, and the color represents the time of publication; the earliest nodes are colored purple while the newest nodes are colored yellow, and thicker lines indicate a higher intensity of collaboration.

An analysis of the citations of the journals revealed that among the top 10 journals in terms of the number of publications (**Table 4**; **Figure 6**), the journal with the largest number of publications was “Plos One”, 2022, IF=3.7, Q2, with a total of 27 articles and a total of 688 citations, and its time of publication in this field was mainly concentrated around 2014. The second most published journal is “Annals of Surgical Oncology”, 2022, IF=3.7, Q2, with 16 articles and 483 citations, and its publications in this field are mainly concentrated around 2019. The journal with the highest average number of citations is the publication “Biomaterials”, IF=14.0, Q1, a total of 13 articles, with an average number of citations of 707 times. An analysis of the literature published in the journal reveals a primary focus on research and advancements in biomaterials. Fluorescence imaging, which uses fluorescent signals emitted by substances upon excitation for imaging, is deeply rooted in biomaterials research and development. The field of biomaterials offers a critical platform that enables medical, biomedical, and life science researchers to disseminate their innovative findings and discoveries.

**Table 4:** Top 10 journals in terms of number of articles issued.

Journal	Articles	Citation	Average Article Citations	IF (2022)	Quatile (2022)
Plos One	27	688	25.48	3.7	Q2
Annals Of Surgical Oncology	16	483	30.19	3.7	Q2
Anticancer Research	13	227	17.46	2.0	Q4
Biomaterials	13	707	54.38	14.0	Q1
Surgical Endoscopy And Other Interventional Techniques	13	105	8.08	3.1	Q2
Molecular Imaging And Biology	12	176	14.67	3.1	Q2
Analytical Chemistry	10	272	27.20	7.4	Q1
Biomaterials Science	10	128	12.80	6.6	Q1
Oncotarget	10	238	23.80	/	/
Diabetologia	9	238	26.44	8.1	Q1

### 3.5 Publication analysis based on highly cited articles

In order to further explore the trend of this field, we analyzed the total citation counts (TCS) and local citation counts (LCS) of 913 papers, as shown in **Tables 5** and **6**. The paper by Welsher K, titled “Deep-tissue anatomical imaging of mice using carbon nanotube fluorophores in the second near-infrared window,” holds the highest TCS with a total of 688 citations and an average of 52.92 citations per year. This study (17) explores the use of well-functionalized biocompatible single-walled carbon nanotubes (SWNTs) as NIR II fluorescent imaging agents for real-time, high-frame-rate video imaging of mice, which allowed observation of SWNTs circulating in the lungs, kidneys, spleen, and liver. Furthermore, dynamic contrast-enhanced imaging through principal component analysis (PCA) enabled the differentiation of organs, including the pancreas. Employing *in vivo* fluorescence imaging in the NIR-II region in conjunction with PCA may provide a robust method for high-resolution optical imaging of deep tissues, relevant for a broad scope of applications spanning biomedical research to disease diagnosis. LCS reflects the citation rate of research among peers. A high LCS indicates that the study has a high degree of peer recognition. The highest LCS is attributed to the paper by Kaushal, Sharmeela et al., “Fluorophore-conjugated anti-CEA antibody for the intraoperative imaging of pancreatic and colorectal cancer,” published in 2008, which has garnered significant academic interest. This study (18) examines how fluorophore-conjugated anti-CEA antibodies enhance cancer visualization in human colorectal and pancreatic cancer models in nude mice, introducing an innovative intraoperative imaging method that has received broad scholarly recognition.

**Table 5:** Top 10 locally cited articles.

Document	LCS	GCS	Normalized LGS	IF (2022)
Kaushal, S, Fluorophore-conjugated anti-CEA antibody for the intraoperative imaging of pancreatic and colorectal cancer. J GASTROINTEST SURG. 2008;	26	116	15.36	3.2
Metildi, CA, Fluorescence-guided surgery with a fluorophore-conjugated antibody to carcinoembryonic antigen (CEA), that highlights the tumor, improves surgical resection and increases survival in orthotopic mouse models of human pancreatic cancer. ANN SURG ONCOL. 2014	17	64	12.47	3.7
Metildi CA, Fluorescently labeled chimeric anti-CEA antibody improves detection and resection of human colon cancer in a patient-derived orthotopic xenograft (PDOX) nude mouse model. J Surg Oncol. 2014;	13	117	9.53	2.5
Tran Cao HS, Tumor-specific fluorescence antibody imaging enables accurate staging laparoscopy in an orthotopic model of pancreatic cancer. Hepatogastroenterology. 2012	12	52	17.78	/
Maawy AA. Comparison of a chimeric anti-carcinoembryonic antigen antibody conjugated with visible or near-infrared fluorescent dyes for imaging pancreatic cancer in orthotopic nude mouse models. J Biomed Opt. 2013	12	32	15.60	3.5
Hoogstins CES, Image-Guided Surgery in Patients with Pancreatic Cancer: First Results of a Clinical Trial Using SGM-101, a Novel Carcinoembryonic Antigen-Targeting, Near-Infrared Fluorescent Agent. Ann Surg Oncol. 2018	11	88	14.37	3.7
Speier S, Noninvasive *in vivo* imaging of pancreatic islet cell biology. Nat Med. 2008	10	201	5.91	82.9
Hiroshima Y,Hand-held high-resolution fluorescence imaging system for fluorescence-guided surgery of patient and cell-line pancreatic tumors growing orthotopically in nude mice. J Surg Res. 2014;	10	62	7.33	2.2
Tummers WS. Intraoperative Pancreatic Cancer Detection using Tumor-Specific Multimodality Molecular Imaging. Ann Surg Oncol. 2018	9	112	11.76	3.7
Newton AD, Intraoperative Near-infrared Imaging Can Identify Neoplasms and Aid in Real-time Margin Assessment During Pancreatic Resection. Ann Surg. 2019	8	47	22.40	9.0

**Table 6:** Top 10 cited articles globally.

Document	TC	TC per Year	Normalized TC	IF (2022)
Welsher K,Deep-tissue anatomical imaging of mice using carbon nanotube fluorophores in the second near-infrared window. Proc Natl Acad Sci U S A. 2011	688	52.92	12.90	11.2
Landen CN Jr, Therapeutic EphA2 gene targeting *in vivo* using neutral liposomal small interfering RNA delivery. Cancer Res. 2005	550	28.95	5.62	11.2
Nagino M, Two hundred forty consecutive portal vein embolizations before extended hepatectomy for biliary cancer: surgical outcome and long-term follow-up. Ann Surg. 2006	392	21.78	5.92	9.0
Komatsu K, Selective zinc sensor molecules with various affinities for Zn2+, revealing dynamics and regional distribution of synaptically released Zn2+ in hippocampal slices. J Am Chem Soc. 2005	345	18.16	3.52	15.0
Yong KT, Imaging pancreatic cancer using bioconjugated InP quantum dots. ACS Nano. 2009	297	19.80	6.09	17.1
Chimienti F, *In vivo* expression and functional characterization of the zinc transporter ZnT8 in glucose-induced insulin secretion. J Cell Sci. 2006	267	14.83	4.03	4.0
Kumar R, Covalently dye-linked, surface-controlled, and bioconjugated organically modified silica nanoparticles as targeted probes for optical imaging. ACS Nano. 2008	259	16.19	4.80	17.1
Kang H, Size-Dependent EPR Effect of Polymeric Nanoparticles on Tumor Targeting. Adv Healthc Mater. 2020	221	55.25	14.54	10.0
van der Vorst JR, Near-infrared fluorescence-guided resection of colorectal liver metastases. Cancer. 2013	220	20.00	8.52	6.2
Fujiwara K, Oleic acid interacts with GPR40 to induce Ca2+ signaling in rat islet beta-cells: mediation by PLC and L-type Ca2+ channel and link to insulin release. Am J Physiol Endocrinol Metab. 2005	213	11.21	2.18	5.1

### 3.6 Keyword-based analysis of publications

**Figure 7:**
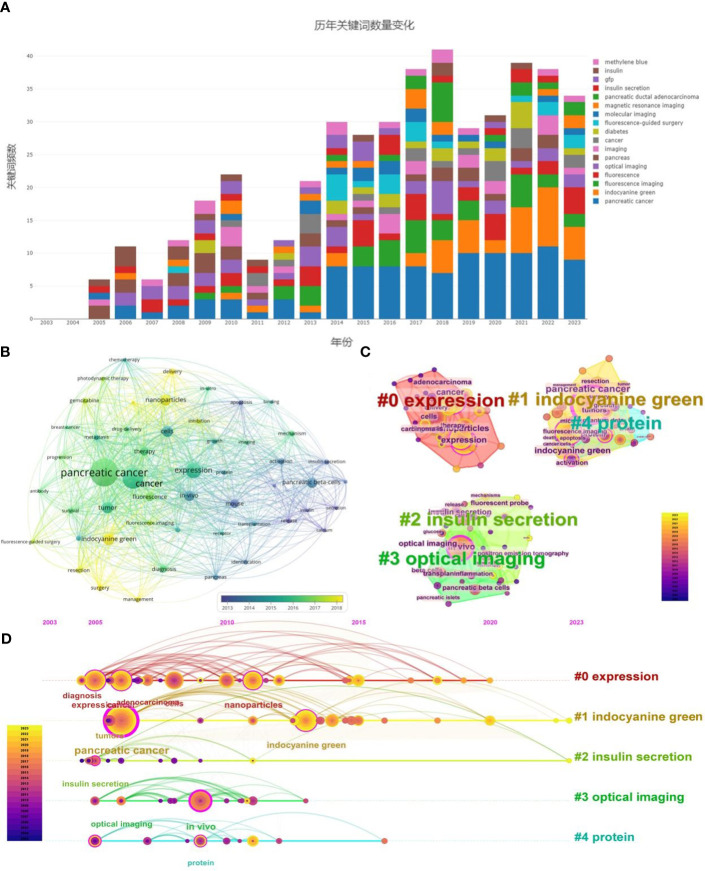
**(A)** Changes in the number of keywords over the years. **(B)** Keyword Network Co-Occurrence Graph: keywords exceeding 20 occurrences are depicted in co-occurrence networks via VOSviewer, where node size correlates with occurrence frequency, and color coding denotes the year of occurrence. Darker hues indicate earlier occurrences, whereas lighter hues signify more recent occurrences. **(C)** Keyword clustering graph: employing CiteSpace, the clustering of the top 10% most frequent keywords is visualized, where node size indicates occurrence frequency, and keywords are clustered based on the LLR algorithm. Each color represents a cluster type, with cluster names determined accordingly. **(D)** Keyword Timeline Chart: keywords generated via CiteSpace are predominantly clustered in a timeline view, with varied colors delineating distinct years and the highlighted lines represent the main concentration time of the clusters. Node size reflects keyword frequency, the chronogram displays the years of keyword emergence, and the right side lists cluster names as determined by the LLR algorithm, providing a comprehensive overview of the research field’s evolution.

Keywords condense the core and essence of a paper, and keyword co-occurrence analysis can discover the research hotspots in a scientific field (**Figure 7A**). We employed VOSviewer to create keyword density and clustering maps for 913 papers, selecting 51 keywords with frequencies of 20 or higher for visualization, as depicted in **Figure 7B**. In this co-occurrence analysis, the size of each node corresponds to the frequency of the topic or keyword, while the length and thickness of the lines between nodes indicate the strength of relationships among keywords. Notably, The significance of a node within a network is directly proportional to the prominence of keyword co-occurrence frequency and centrality. **Figure 7B** reveals that ‘pancreatic cancer’ (n=174, centrality=0.44), ‘cancer’ (n=130, centrality=0.16), ‘expression’ (n=96, centrality=0.19), ‘indocyanine green’ (n=72, centrality=0.10), ‘tumor’ (n=70, centrality=0.05), ‘cells’ (n=69, centrality=0.07), ‘nanoparticles’ (n=68, centrality=0.12), ‘pancreatic beta-cells’ (n=65, centrality=0.06), ‘*in-vivo*’ (n=64, centrality=0.21), ‘therapy’ (n=55, centrality=0.01), among other high-frequency keywords, are the predominant terms in this field. The co-occurrence diagram analysis shows a central focus on keywords like ‘indocyanine green’ and ‘nanoparticles’ around 2020, indicating a recent concentration in research within this area. Additionally, ‘pancreatic cancer’ has been a principal keyword since 2014, consistently receiving significant attention from scholars.

The analysis of keyword centrality conducted using CiteSpace revealed that ‘pancreatic cancer’ (0.44), ‘*in-vivo*’ (0.21), ‘cancer’ (0.16), ‘expression’ (0.19), and ‘insulin secretion’ (0.15) were among the keywords with the highest centrality. These terms play a pivotal role in driving the advancement and development of research within the discipline.

High-frequency co-occurring keywords were extracted using CiteSpace, yielding a total of 136 keywords. These were categorized into five clusters for analysis, as shown in **Figure 7C**: “#0 expression” (red), “#1 indocyanine green” (yellow), “#2 insulin secretion” (green), “#3 optical imaging” (grass green), and “#4 protein” (cyan). Modularity (Q value) indicates the strength of the clustering structure; a Q value greater than 0.3 is generally considered significant. Weighted Mean Silhouette (S value) assesses the clustering validity; an S value above 0.5 suggests reasonable clustering, and above 0.7 indicates convincing clustering. Clusters are enumerated from 0 to 5, with a smaller number representing a larger number of contained keywords, and each cluster is composed of multiple closely related terms. The CiteSpace Keyword Clustering Network Knowledge Graph categorizes keywords into five clusters, with a modularity value of 0.5183 and a silhouette score of 0.8026. These values indicate a highly significant clustering structure and a convincing clustering effect. Specifically, the ‘expression’ theme demonstrates a notable concentration, encompassing keywords such as ‘expression’, ‘diagnosis’, ‘treatment’, ‘adenocarcinoma’, ‘nanoparticles’, and ‘delivery’. Additionally, fluorescence imaging plays a crucial role in real-time observation and recording of protein or other biomolecular expression dynamics. This technique is vital for understanding cellular processes, signaling pathways, and biological mechanisms, which underscores its prominence in the field.

Keyword co-occurrence analysis sheds light on the foundational elements, prevalent themes, and focal points of a research domain, while evolution analysis of keywords elucidates the developmental trajectory of the domain. In this context, the present study employs CiteSpace for evolutionary analysis of keywords, including a keyword timeline analysis, with the latter integrating a temporal dimension into the clustering view. This diachronic perspective on keyword evolution illuminates shifting trends and alters the domain’s emphases over time. Applying the Log-Likelihood Ratio (LLR) algorithm to cluster and designate prominent thematic categories, **Figure 7D** reveals that from 2005 to 2010, research predominantly focused on the application of fluorescence imaging technology in pancreatic diseases, particularly pancreatic cancer, with keywords such as ‘expression,’ ‘proteins,’ ‘*in vivo*,’ ‘pancreatic cancer,’ ‘diagnostic,’ and ‘therapeutic’ to the fore. Subsequently, from 2010 to 2015, the emphasis shifted to the research and development of specific molecular probes, exemplified by terms like ‘indocyanine green’ and ‘nanoparticles,’ a focus that has persisted over subsequent years.

**Figure 8:**
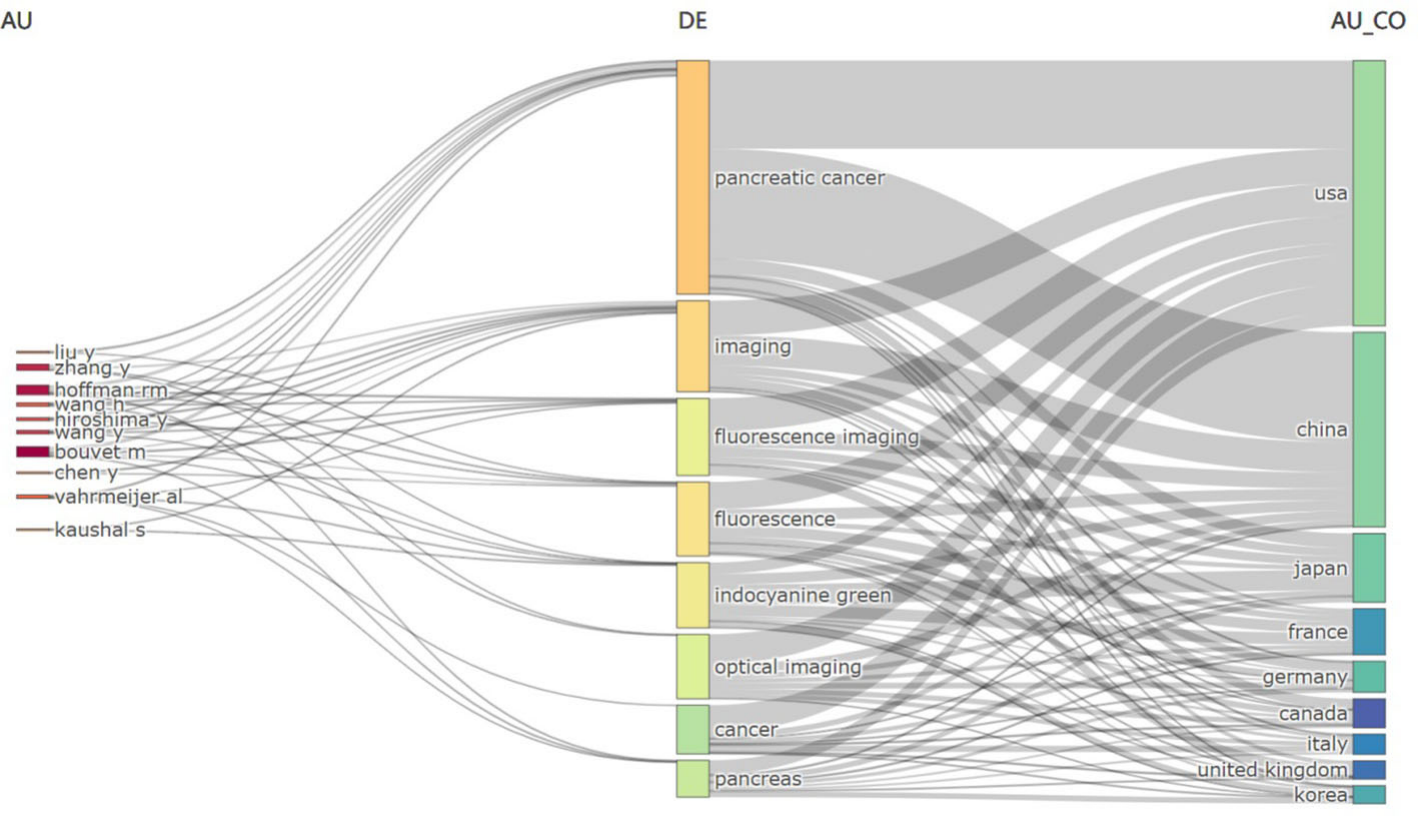
Three fields plot: Relationships between authors (left), keywords (center) and countries (right) in the field were processed using Bibliometrix: R-tool version 4.1.4.

**Figure 8** presents a comparative analysis of the top 10 authors, keywords, and countries in terms of research intensity, as depicted in the Three Fields Plot. In this plot, the size of the components correlates with the degree of interrelation, with larger components indicating closer relationships. The higher positioning of a rectangle within the plot reflects a greater degree of relevance. The analysis indicates a significant concentration of research efforts in the United States, China, and Japan, particularly focusing on topics such as “pancreatic cancer,” “imaging,” “fluorescence imaging,” “fluorescence,” and “indocyanine green.” Notably, the volume of research on pancreatic cancer appears more substantial in China than in the United States.

**Figure 9:**
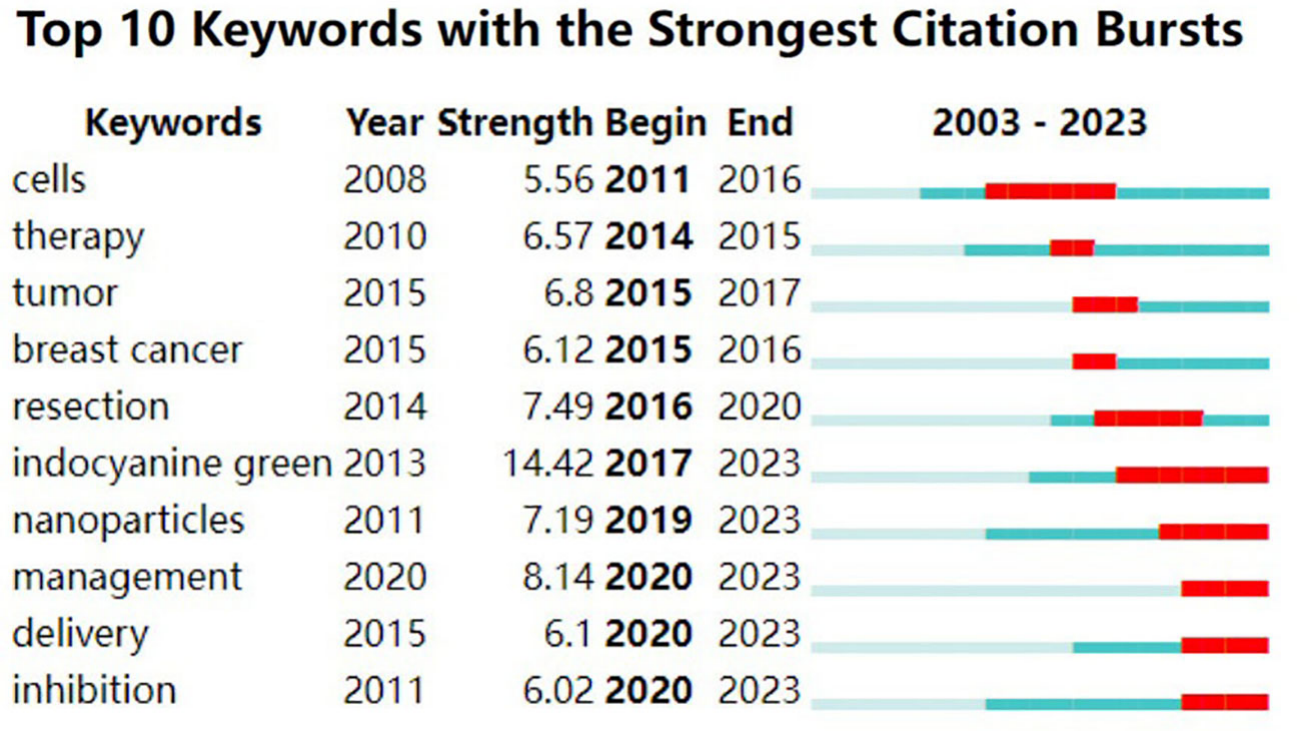
Top 10 Keywords with the Strongest Citation Bursts: burst word analysis using Citespace. On the right is a timeline view of the emergence of the burst words, with red indicating the time of the keyword burst, blue indicating the time period of existence, and “Strength” indicating the strength of the burst word.

A ‘burst word’ refers to a keyword that exhibits a significant increase in frequency over a short period. The detection of keyword bursts serves as an indicator of research frontiers or emerging topics in a specific field over time. **Figure 9** highlights the top 10 keywords with the strongest emergence intensity. In this figure, ‘Year’ indicates the year of the keyword’s initial appearance; ‘Begin’ and ‘End’ denote the start and end years of the keyword’s burst, respectively. ‘Strength’ represents the intensity of the keyword’s burst, with higher values signifying greater importance. Notably, ‘indocyanine green’ emerges as a leading research frontier for the period 2017-2023, lasting for 6 years with a burst strength of 14.42, making it the keyword with the highest strength. Recent years have seen ‘indocyanine green’, ‘nanoparticles’, ‘management’, ‘delivery’, and ‘inhibition’ as prominent research frontiers.

### 3.7 Publication analysis based on co-citations

**Figure 10:**
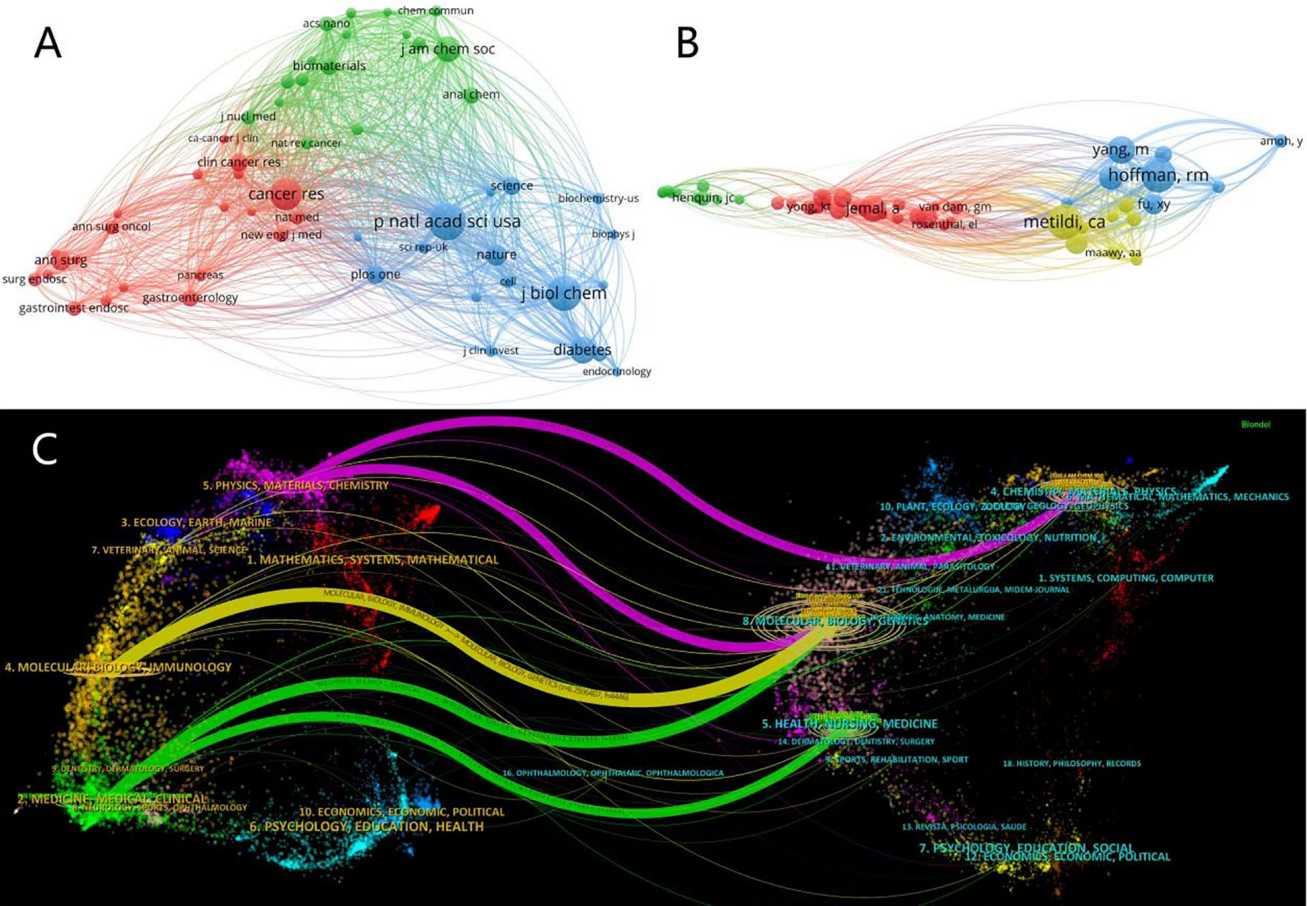
**(A)** Co-Cited Journals Network Co-occurrence Graph: co-occurrence network visualization of journals with more than 130 citations using Vosviwer. Larger nodes indicate more citations, colors represent clustering, and thicker lines indicate higher collaboration intensity. **(B)** Co-Cited Author Network Co-Occurrence Graph: Co-cited authors Co-occurrence network visualization of authors with more than 21 citations using Vosviwer. Larger nodes indicate more citations, colors represent clustering, and thicker lines indicate higher collaboration intensity. **(C)** The dual-map overlay: use CiteSpace to visualize the mutual citation between periodicals. On the left is the type of cited journals, while on the right is the type of cited journals; the lines represent the cited relationship between them. The more papers published in the journal, the longer the vertical axis of the ellipse, the more the number of authors, the longer the horizontal axis of the ellipse.

The objective of co-citation analysis is to identify frequently cited papers and the journals in which they are published within a specific research field. Utilizing VOSviewer, a co-citation map of journals was constructed. The threshold for inclusion was set at a minimum of 130 co-citations, resulting in a selection of 55 journals for the co-citation analysis. The resultant co-citation relationship map is presented in **Figure 10A**. The co-citation network of journals is depicted in the figure, comprising three clusters distinguished by three distinct colors. The network features the top three highly cited journals: ‘Proceedings of the National Academy of Sciences of the United States of America’ (840 citations), an interdisciplinary journal of high repute that publishes transformative research with broad implications for the scientific community and society, fostering interdisciplinary collaboration, including in the fields of biology and medical research; ‘Journal of Biological Chemistry’ (777 citations), which showcases pivotal biochemistry research articles, advancing cutting-edge research in biochemistry, contributing significantly to the scientific community’s in-depth understanding of life molecules and their functions; and ‘Cancer Research’ (710 citations), with a primary focus on oncological studies. Pancreatic cancer is the third most common malignant tumor worldwide Thus, contributions to this journal are strongly reflected in the citation patterns within our area of investigation. The three journals highlighted are esteemed in their respective fields, with an average Impact Factor (IF) for 2022 of 9.03. Within the three clusters of the co-citation network, the journals in the green cluster predominantly represent the disciplines of chemistry, biochemistry, and materials science. This cluster emphasizes the crucial role of fluorescence imaging, particularly in the application of specific stains in living organisms, which is integral to the fields of chemistry, materials science, and biochemistry. The citations of these journals primarily serve to analyze and review existing research, offering theoretical and empirical support for further studies. The blue cluster encompasses journals from interdisciplinary and multidisciplinary fields, including eminent publications like ‘Nature’ and ‘Science’. Journals in the red cluster are primarily focused on medical subjects, covering areas such as the digestive system, pancreas, oncology, and surgery. These publications are noted for their emphasis on the technical aspects of data collection, clarity, and analysis, and are cited principally to provide technical insights into research.

Among the leading co-cited authors (**Figure 10B**), the most frequently cited is Metildi, C.A. (h-index(Wos)**=**13). Although the number of publications attributed to Metildi is limited, the high quality of his work is universally acknowledged within the field. Notably, two of his articles are listed in the local TOP10 for citations (19, 20). His pivotal research introduced a carcinoembryonic antigen (CEA) fluorophore-linked antibody, advancing fluorescence-guided surgery techniques for pancreatic cancer in murine models. This contribution has garnered considerable attention and acclaim, being cited 106 times in our research domain. Following closely, the second most cited author is Hoffman, Robert M. (h-index(Wos)=76). Hoffman holds the distinction of having the highest publication count and enjoys profound recognition in the field, leading with an impressive total of 104 citations.

Five major citation pathways were identified: two in green, one in yellow, and two in purple (**Figure 10C**). The yellow pathway signifies that publications from the Molecular/Biology/Genetics journals are predominantly cited by those in the Molecular/Biology/Immunology field. The green pathway reveals a trend where publications in Molecular/Biology/Genetics and Health/Nursing/Medicine are frequently cited by those in Medicine/Medical/Clinical journals. The purple pathway highlights that publications in Molecular/Biology/Genetics and Chemistry/Materials/Physics are most often cited by works in the Physics/Materials/Chemistry domain.

To further explore the developmental trends in this field, we conducted an analysis of the references cited in the selected literature. Utilizing VOSviewer, we examined the top five cited works in this field from 2003 to 2023, as detailed in the **Table 7**. The table reveals that the most frequently cited document is ‘Cancer Statistics 2009’, indicating a predominant focus on pancreatic cancer within the research on fluorescence imaging technology for pancreatic diseases. The subsequent three articles primarily discuss the combination of fluorescent dyes with specific targeting mechanisms for tumor visualization, contributing to advancements in intraoperative imaging technology. The fifth article addresses the development of an *in situ* mouse model of pancreatic cancer, which faithfully replicates the invasive process of human pancreatic cancer, thereby laying a crucial foundation for future research in this domain. This analysis of highly cited references underscores the significant emphasis on pancreatic cancer in the context of fluorescence imaging, highlighting it as a critical area of focus and underscoring the urgent need for further research to address clinical challenges.

**Table 7:** Top 5 cited references.

References	citations
Jemal A, Cancer statistics, 2009. CA Cancer J Clin. 2009	39
van Dam GM, Intraoperative tumor-specific fluorescence imaging in ovarian cancer by folate receptor-α targeting: first in-human results. Nat Med. 2011	32
Kaushal S, Fluorophore-conjugated anti-CEA antibody for the intraoperative imaging of pancreatic and colorectal cancer. J Gastrointest Surg. 2008	26
McElroy M, Imaging of primary and metastatic pancreatic cancer using a fluorophore-conjugated anti-CA19-9 antibody for surgical navigation. World J Surg. 2008;	25
Katz MH, A novel red fluorescent protein orthotopic pancreatic cancer model for the preclinical evaluation of chemotherapeutics. J Surg Res. 2003	24

**Figure 11:**
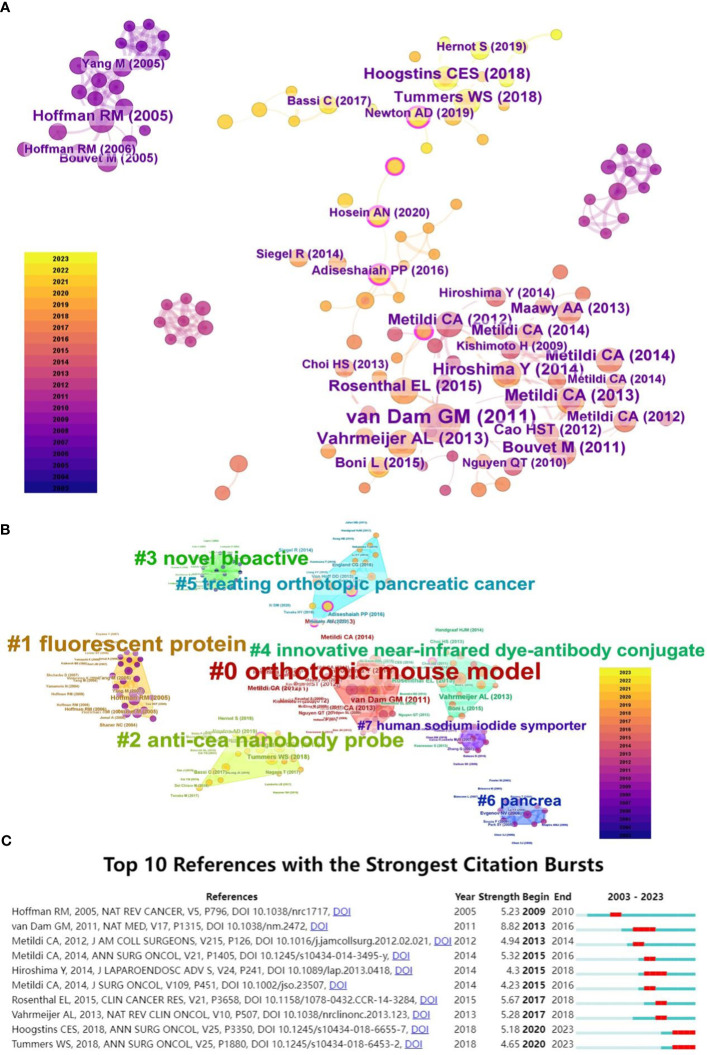
**(A)** Co-Cited Articles Network Co-occurrence Graph: set g-index (k=5) with CiteSpace to visualize the cited articles in the network, each node represents an article, the node size represents the number of citations, and the rings of different widths of nodes represent different years to be cited. The color of the node connection indicates the year in which it is co-cited, and the thickness indicates the number of times it has been cited. **(B)** Cited Articles Clustering Graph: using Citespace to set g-index (k=5), the cited references are clustered and visualized, each node represents an article, and the node size represents the number of cited articles. Based on the LLR algorithm, the cited articles are clustered, each color represents a cluster, and the cluster name is calculated. **(C)** Top10 References with the Strongest Citation Burst: Citespace was used to analyze co-cited references. On the right is the timeline view of the emergence of the article. Red indicates the time when the article broke out, blue indicates the period of existence, and “Strength” indicates the intensity of the emerging word.

A total of 217 references were extracted from the corpus of cited literature and subsequently subjected to a clustering analysis using CiteSpace, as depicted in **Figures 11A, B**. This analysis yielded eight distinct clusters, with a modularity value (Q) of 0.8715 and a silhouette score (S) of 0.9804, denoting highly significant structural integrity and a convincing clustering effect. The clusters are identified as follows: “#0 Orthotopic Mouse Model”, “#1 Fluorescent Protein”, “#2 Anti-CEA Nanobody Probe”, “#3 Novel Bioactive”, “#4 Innovative Near-Infrared Dye-Antibody Conjugate”, “#5 Treating Orthotopic Pancreatic Cancer”, “#6 Pancrea”, and “#7 Human Sodium Iodide Symporter”. The order of these clusters indicates the degree of similarity among the references they contain, with each cluster encompassing multiple interrelated references. Notably, the clusters pertaining to “Orthotopic Mouse Model”, “Fluorescent Protein”, and “Anti-CEA Nanobody Probe” stand out as the three most frequently cited.

The analysis indicates that from 2013 to 2016 (**Figure 11C**), the leading research frontier was defined by the publication of Van Dam GM et al., titled “Intraoperative tumor-specific fluorescence imaging in ovarian cancer by folate receptor-α targeting: first in-human results.” During this brief span of three years, the emergence of related themes reached a zenith with a word intensity of 8.82. This seminal work (21) illustrated the efficacy of utilizing specialized fluorescent probes in intraoperative tumor-specific fluorescence imaging for ovarian cancer patients, thereby providing invaluable insights to a broad spectrum of researchers. In more recent years, a pivotal article by Hoogstins CES et al., published in 2018, “Image-Guided Surgery in Patients with Pancreatic Cancer: First Results of a Clinical Trial Using SGM-101, a Novel Carcinoembryonic Antigen-Targeting, Near-Infrared Fluorescent Agent,” marked a trend with an intensity of 5.18. This study (22) presents a clinical trial evaluating a novel, fluorescently labeled anti-CEA antibody for intraoperative visualization in pancreatic cancer surgery, triggering an increased interest in clinically applying specific probes beyond traditional fluorescent agents in humans. Additionally, Tummers WS et al.’s 2018 publication “Intraoperative Pancreatic Cancer Detection using Tumor-Specific Multimodality Molecular Imaging” revealed an intensity of 4.65. This article (23) reported the inaugural application of multimodal molecular imaging using cetuximab-IRDye800 in both primary and metastatic human pancreatic tumors for intraoperative assays, successfully confirming the technique’s efficacy and safety.

## 4 Discussion

Effective knowledge management is increasingly recognized as an essential tool for researchers to stay current with the latest advancements in their fields. Systematic and quantitative analysis of the literature is indispensable in fostering the growth of a research domain, as well as a critical method for the academic community to gain a comprehensive understanding of research trends, outcomes, prominent institutions, and collaborative patterns in the field of fluorescence imaging of the pancreas. With the rapid progress in information technology, the topic has garnered widespread interest and prompts continual updates in the research agenda. In this study, we conducted a systematic search of the Web of Science (core collection) for articles published between January 1, 2003, and December 1, 2023, on pancreatic fluorescence imaging. This search yielded 913 publications that satisfied the inclusion criteria. Using VOSviewer, R language, and CiteSpace software, we performed an in-depth analysis of two decades of research in this area. Our review offers a systematic overview of field development, identifies leading national institutions, key contributors, principal journals, influential publications, central keywords, and significant references, and synthesizes the conclusions derived from bibliometric analyses.

Collectively, the volume of articles in this field is on the ascent, with a notable surge observed post-2014. The increase in published articles signifies a growing emphasis on this field by clinicians and researchers. Furthermore, there is a discernible trend towards enhanced collaboration among authors, institutions, and countries, underscoring the escalating attention and limitless research prospects within this domain. The forefront of contributions originates predominantly from the United States, China, and Japan, each demonstrating substantive collaborative endeavors. The United States leads in both the quantity and impact of publications—with the highest centrality and average citation count—evidencing its dominant role in advancing this domain. In addition, the United States is home to four of the top ten most prolific institutions and seven of the top ten leading authors, further attesting to its influential position. Meanwhile, China holds the position of second-most prolific contributor, with its publication output showing a continuous upward trajectory in recent years. Post-2020, the publication output from China has surpassed that of the United States, embodying a significant presence in the field with a higher centrality. However, despite this prolific output, Chinese publications register fewer citations. For China to augment its scholarly impact, there is a necessity to shift focus from quantity to quality. Emphasizing academic innovation and fostering greater international collaboration with scholars from diverse nations will be seminal for enhancing its scientific standing. Such strategic efforts are poised to elevate the professional stature within the field and enable more profound contributions to the exploration of research frontiers.

The University of California, San Diego, and AntiCancer Inc. are the two most cited organizations in this field. Notably, four of the top ten scholars, including Hoffman, Robert M., Bouvet, Michael, and Kaushal, Sharmeela, among others, demonstrate close collaboration with one another and have co-authored numerous publications. Their work has contributed indelibly to the discipline’s development and progress. Among them, Hoffman, Robert M. is the scholar with the most published articles (n=99, h-index (WoS) = 76), boasting an average citation count of 32.0. An eminent scholar, he has contributed to diverse fields, ranging from oncology to cell biology, biochemistry, molecular biology, and surgery. Since 2005, his research focus has been on the application of fluorescence imaging technology in pancreatic studies, where his work plays a pivotal role in pioneering this field’s research. Massachusetts General Hospital is the institution with the highest citation tally. These findings collectively affirm the United States’ predominant influence in the field, not solely due to its considerable population and substantial financial resources for the scientific community, but also owing to its cultural traits that emphasize innovation, which are imperative factors. The co-authorship, institutional and country collaboration networks, and author co-citation analysis graphs illustrate that authors within the same research area are interconnected, as are their respective studies. Collaboration also thrives among authors from varied research areas, with their studies being complementary and mutually reinforcing. Consequently, fostering enhanced collaboration among principal investigators across various research domains is anticipated to ignite novel breakthroughs in the discipline.

The analysis of academic journals not only aids scholars in selecting the most suitable venues for their research dissemination, but also facilitates a more comprehensive understanding of the influential journals within the discipline, thereby enhancing opportunities to access cutting-edge advancements. “Plos One” and “Annals of Surgical Oncology” are the two journals with the highest publication counts, indicating their prominent interest in this realm of research; yet the average citation numbers and impact factors (IF) are relatively low. Regarding research quality, only four journals within the top ten have an IF exceeding 5. As the application of fluorescence imaging technology in pancreatic studies is still an emerging research field and intersects multiple disciplines and domains, this area of research has not yet received substantial attention. This results in challenges for articles in this field to be featured in high-impact journals. However, with the expanding scope and depth of research in recent years, it has the potential to emerge as a focal point in high-impact journals. The journal Biomaterials holds the highest impact factor (IF) at 14.0 and also leads in the highest average citation count, as the citation count further reflects the journal’s influence, suggesting that research published in these journals tends to garner more attention.

Citation count is a critical metric for assessing the academic impact of a publication, and this study identifies the top 10 papers in this domain based on local citation counts (LCS) and total citation counts (TCS). The LCS measures the citation frequency within the scientific community, and a high LCS indicates substantial peer recognition of the research. The majority of the top 10 LCS-ranked articles concentrate on the following themes (18, 20–28): (1) The use of specific fluorescent agents, particularly those conjugated with anti-CEA antibodies for tumor targeting, has emerged as a research trend, facilitating fluorescence imaging of pancreatic tumors, including applications in surgical navigation, tumor evaluation, margin assessment, and tumor staging. These applications extend from animal models to human clinical use and have yielded objective results. (2) *In vivo* fluorescence imaging of pancreatic islet cells is another focal area, laying the foundation for clinical advancements in pancreatic diseases and introducing novel concepts. Among the top 10 articles by total citation counts (TCS), Kevin Welsher et al. (17) examined the use of near-infrared dual-region imaging combined with deep tissue probing, which established a foundation for subsequent pancreatic applications in this domain. Landen CN Jr et al. (29) evaluated the effectiveness of targeted probes in drug delivery. With additional insights into specialized probes and diverse mechanisms and pathways, these studies have established an important groundwork for the field’s development and have offered direction and impetus for applying fluorescence imaging technology in pancreatic research. Concurrently, they enhance our understanding of advancements within this discipline.

Keywords serve as the essential summaries of scholarly research. By analyzing the keywords prevalent at various stages, it becomes possible to track the forefront of advancements in this discipline, to discern the research trends within specific timeframes or contexts, as well as to capture the overarching development of the field in recent years, thereby guiding future research endeavors. At present, in the field of fluorescence imaging technology in the pancreas, it can be seen from the co-occurrence chart that pancreatic cancer is still the most concerned term in this field (n=174 centrality=0.44), which has been present since its appearance in 2006. This is followed by terms like ‘cancer’, ‘tumor’, among others, also ranking highly, illustrating the broad applicability of fluorescence imaging technology in various cancer types, including breast, gastric, and ovarian cancer. Over the past several decades, a multitude of fluorescence imaging techniques have been developed with the goal of achieving precise tumor elimination while maximizing the preservation of organ function. During oncologic surgery, fluorescence imaging can visualize clinically occult lesions and adjacent critical structures with heightened sensitivity and resolution. This provides a safe and effective real-time imaging solution to clinical challenges, enhancing the quality of surgical interventions and improving patient outcomes. This highlights its significant research importance and practical value, offering a safe and effective real-time imaging solution for clinical challenges. Additionally, terms like ‘indocyanine green’ and ‘nanoparticles’ have gained prominence since around 2020, indicating a recent concentration of research focus in this area. Analysis of bursts words also reveals ‘indocyanine green’ as a leading research frontier from 2017-2023, with an intensity of 14.42. Indocyanine green (ICG) is the sole near-infrared imaging agent approved by the FDA for clinical applications. It has an absorption peak between 790 and 810 nm and emits fluorescence in the 820 to 830 nm range. Notably, it is highly safe, exhibits minimal allergic reactions, has a short *in vivo* half-life, and is rapidly cleared, having been approved for clinical use since 1956. ICG is now widely utilized in various medical and biomedical fields, including liver, gallbladder, gastrointestinal, urinary, mammary, and skin flap procedures (30). Recently, the application of ICG in pancreatic cancer treatment has been steadily rising. ICG offers significant benefits in pinpointing pancreatic tumors, detecting micrometastases, assessing peripancreatic lymph nodes, and evaluating tissue perfusion.

However, certain limitations hinder its broader development, which include: I. Difficulty in imaging target tissues or low fluorescence intensity: Target tissues (e.g., tumors, lymph nodes, etc.) may pose challenges for imaging due to insufficient fluorescence intensity or the occurrence of reverse development (negative development). II. Problems with ICG application dose: In liver tumors, the injection dose of ICG is usually 0.25-0.50 g/kg, while in pancreatic tumors, it is 2-5 mg/kg (31). However, excessive doses may lead to serious complications such as anaphylaxis. III. Fibrous stromal barrier around the tumor: Pancreatic tumors, especially pancreatic cancer, are often accompanied by fibrous stromal hyperplasia, which is a natural obstacle to drug delivery (32), hindering ICG penetration into the tumor core. IV. Tumor blood supply problem: Compared with the liver, which has two sets of blood supply, the pancreas is a lack of blood supply organ. Pancreatic cancer lacks blood-supplying arteries, which leads to difficulty in enriching the probe in the tumor tissue. V. Normal tissue imaging problem: Imaging normal tissue carries the risk of false positives, largely due to an inappropriate timing window post-ICG injection or excessive dosing, leading to drug retention in normal tissues. VI. ICG penetration depth problem: Although the depth of ICG in NIR-II(Near-infrared-II) imaging has been improved relative to NIR-I (Near-infrared-I) imaging, it remains inadequate for imaging deep-seated pancreatic tumors (3). VII. Inadequate imaging instrumentation: There is a lack of commercially available instruments for NIR-II fluorescence imaging, so the number of NIR-II fluorescence-guided surgical navigation is low. These issues limit the validity and reliability of ICG in clinical applications and require further research and technological development to address.

Therefore, how to circumvent the above drawbacks and improve the application of fluorescence imaging in pancreatic diseases is a challenge we need to solve. By analyzing the current situation in depth and combining cutting-edge research results and practical experience, this study proposes a few potential solutions: I. Development of targeting probes utilizing ICG properties: Indocyanine green (ICG) has demonstrated considerable potential in the treatment and diagnosis of various human diseases. Despite some limitations in its application to pancreatic diseases, ICG still possesses tremendous potential for development. Future research should focus on leveraging ICG’s characteristics to develop targeted probes (31). By combining ICG with artificially synthesized antibodies against tumor markers, we can enhance the probe’s fluorescence development efficiency and targeting accuracy while ensuring good biocompatibility. Such probes can be directed towards specific biomarkers or tissues for precise imaging, thus enhancing the specificity and accuracy of the technique and delivering more precise information for clinical diagnosis and treatment. Numerous researchers have merged ICG with synthetically engineered tumor marker antibodies, demonstrating favorable pharmacokinetic properties and enhanced target-to-background signal ratios in organs (33). Luo, X et al. (3) developed a dextran-indocyanine green (DN-ICG) nanoprobe in the NIR-II spectrum for dynamic imaging of tumor-associated macrophages (TAMs) in pancreatic cancer. The DN-ICG nanoprobe exhibited a 279% increase in NIR-II fluorescence intensity compared to free ICG, alongside markedly enhanced stability. It was demonstrated that the DN-ICG nanoprobe could selectively target TAMs through dextran’s interaction with ICAM-3-associated protein 1 (SIGN-R1), abundantly expressed in TAMs. Subsequently, in a mouse model, the DN-ICG nanoprobe was gradually metabolized in the liver yet persisted within the pancreatic tumor matrix, achieving a high signal-to-noise ratio (SBR=7) in deep tissue (approximately 0.5 cm) NIR-II imaging of TAMs. The DN-ICG nanoprobe possesses high biocompatibility and biodegradability, indicating significant potential in the precision therapy of pancreatic cancer. Wang, M et al. (34) engineered an amorphous iron oxide nanoparticle platform (ION) tailored to the tumor microenvironment for the co-delivery of photothermal agents (ICG) and toll-like receptor 7 agonists (IMQ). The biodegradable nano-platform, IMQ@IONs/ICG, enhances the permeability of the encapsulated drugs within pancreatic tumors. Results indicated that IMQ@IONs/ICG successfully surmounted the challenge of drug delivery to pancreatic tumors, and, in conjunction with photothermal therapy, was capable of inducing systemic anti-tumor immunity to manage metastatic tumors. Qi et al. (35) synthesized hyaluronic acid-based nanoparticles integrated with ICG, creating NanoICG for intraoperative near-infrared fluorescence detection in pancreatic cancer. In an *in situ* pancreatic ductal adenocarcinoma model treated with NanoICG, significant fluorescence accumulation was observed in the pancreas, demonstrating enhanced visibility of pancreatic lesions compared to non-diseased pancreatic tissue. Compared to ICG injection, fluorescence microscopy revealed greater fluorescence intensity of NanoICG in pancreatic lesions and splenic metastases. In the future, transitioning ICG-targeted and other fluorescent-targeted drugs to clinical use is expected to significantly advance fluorescence imaging. As fluorescence imaging technology continues to evolve, ICG is poised to play an increasingly vital role in diagnosing and treating pancreatic cancer, and this will likely enhance the effectiveness and prognosis of pancreatic cancer surgeries. II. Optimization of NIR emitters: Most NIR emitters are still in the preclinical stage, and a portion of NIR-I imaging techniques have been applied with clinical applications, but there is still a dearth of commercial instruments for NIR-II imaging. Compared with NIR-I (700-900 nm), NIR-II (1,000-1700 nm) imaging exhibits reduced autofluorescence interference, enhanced tissue penetration, and an elevated signal-to-background ratio; consequently, NIR-II fluorescence imaging holds the potential to enhance the diagnosis of deep-seated diseases and represents a promising direction for the future of pancreatic fluorescence surgical navigation (36). Consequently, optimizing the current emitters and investigating the creation of novel emitters with outstanding optical and biological characteristics is imperative. III. Development of complex image analysis software: Concurrently, the development of more advanced image analysis software for processing and interpreting data from fluorescence imaging is essential. This advancement will enhance the interpretation of imaging results and the practicality of clinical applications. IV. Multidisciplinary cooperation: Fostering interdisciplinary collaboration is imperative for bridging the gap between preclinical research and clinical practice. The establishment of a multidisciplinary cross-cutting model, the cultivation of talents at the intersection of medicine and science and technology, and the intensification of team collaboration will expedite the translation of technological advances into clinical practice.V. Establishment of effective databases: Establishing comprehensive databases is crucial for advancing technology. These databases serve to store and share imaging data, foster scientific exchange and collaboration, and expedite the development and application of technology. VI. Strengthen collaboration with industry and academic partners: Tight collaboration with industry and academic partners can streamline the translation and commercialization of technologies. Through such synergy, research outcomes can be integrated into clinical practice more swiftly, thereby actualizing the clinical advantages of the technology. These solutions not only help to address current challenges, but also lay a solid foundation for ICG’s long-term development.

Fluorescence molecular imaging has demonstrated considerable promise in aiding the pancreatic diagnostic process and in the potential to enhance clinical outcomes. Despite enthusiasm for these preliminary findings, intraoperative fluorescence molecular imaging stands at a pivotal juncture. To advance beyond proof-of-concept studies and effect a true transformation in clinical practice, rigorous efficacy studies are required. Upon taking this step, it is anticipated that increased investment will flow into the creation of innovative tracers with enhanced pharmacokinetic properties and specificity, alongside the development of advanced image analysis software.

Co-citation analysis reveals the most frequently cited journals and seminal literature in this domain, aiding subsequent researchers in identifying the central research within this field and enabling them to more rapidly understand the core content and comprehensive framework of this field. The co-cited journals’ co-occurrence network diagram illustrates that this interdisciplinary field draws on references from a diverse range of disciplines, including chemistry, biochemistry, materials science, molecular biology, clinical medicine, among others. This demonstrates the field’s comprehensive nature, necessitating full collaboration among scholars from various disciplines. Increased academic exchange is anticipated to foster novel breakthroughs in this area.

Through co-occurrence and cluster analysis visualization of references, the algorithm identifies 8 distinct clusters of references, each cluster being closely related to the field and pivotal in its research history. This analysis facilitates scholars from various disciplines to fully understand the field and rapidly identify research hotspots, cutting-edge technologies, and future directions.

While bibliometric analysis assists to a certain extent in discerning the developmental trends and focal areas of a research field, thereby facilitating researchers in quickly identifying cutting-edge developments, this study is not without its limitations. Initially, it encompasses only English-language articles sourced from the Web of Science Core Collection, which may lead to certain data exclusions. Secondly, the analytical tools employed, namely CiteSpace, VOSviewer, and R Studio, are not equipped to perform full-text analysis, which results in discrepancies in data analysis and the risk of missing crucial information. Thirdly, research is inherently a dynamic and evolving endeavor, characterized by its timeliness. Consequently, our analysis necessitates ongoing improvements in the future.

In summary, the application of fluorescence imaging technology in pancreatic surgery has sparked increasing interest globally, with a growing number of scholars focusing on this domain. This technology has been demonstrated to be safe and effective and is poised to significantly advance pancreatic surgery. However, its clinical application remains in the developmental stage and has not yet fully matured. With ongoing technological advancements, it is anticipated that this technology will see broader future applications, providing better medical services for patients.

## Data availability statement

The datasets presented in this study can be found in online repositories. The names of the repository/repositories and accession number(s) can be found below: https://www.jianguoyun.com/p/Df_7uCcQouSuDBiWqrYFIAA.

## Author contributions

QL: Data curation, Formal analysis, Software, Visualization, Writing – original draft, Writing – review & editing. XT: Data curation, Formal analysis, Software, Validation, Visualization, Writing – review & editing. LZ: Data curation, Formal analysis, Methodology, Validation, Writing – original draft. MD: Investigation, Methodology, Supervision, Validation, Writing – review & editing. KC: Data curation, Investigation, Methodology, Validation, Writing – review & editing. JY: Writing – review & editing, Investigation, Validation. WC: Methodology, Supervision, Validation, Visualization, Writing – review & editing.
