# Supplementary material for: Global trends in the application of fluorescence imaging in pancreatic diseases: a bibliometric and knowledge graph analysis
Source: Front Oncol. 2024 Jul 19;14:1383798. doi: 10.3389/fonc.2024.1383798 (PMC11294181; doi:10.3389/fonc.2024.1383798)
Supplement: Supplementary file 1 [file DataSheet_1.docx]

Supplementary Material

# Supplementary Figures


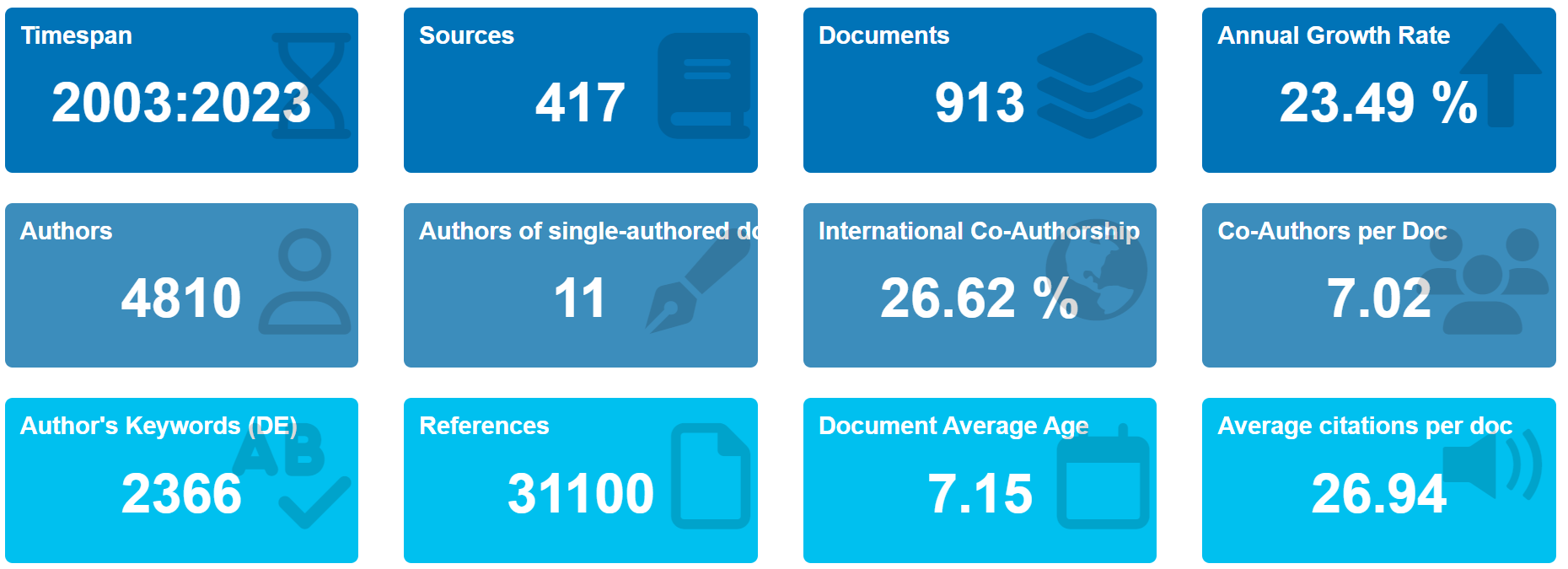


**Supplementary Figure 1.** Visualizing an overview of information in this field using Bibliometrix: R-tool version 4.1.4


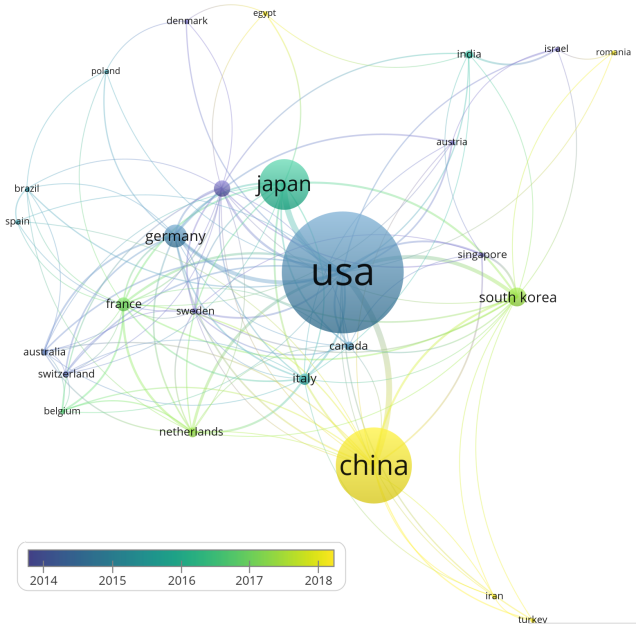

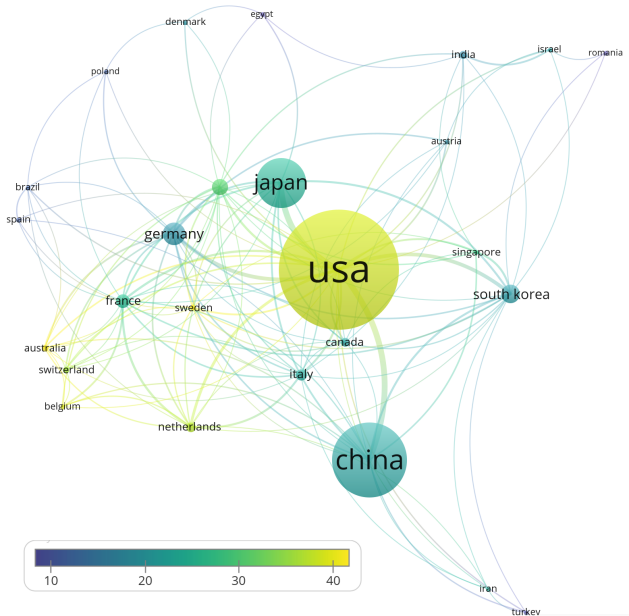


**Supplementary Figure 2.** Country Cooperation Network Co-occurrence Graph： VOSviewer visualizes countries that have published more than or equal to 5 articles. Each node represents an institution, the size of the node represents the number of publications, and the annual rings of different widths of the nodes represent articles published in different years. (a) The color of the node represents the time of publication. The darker the color, the earlier the time. (b) The color of the node represents the number of citations per article. The darker the color, the fewer the number of citations per article.


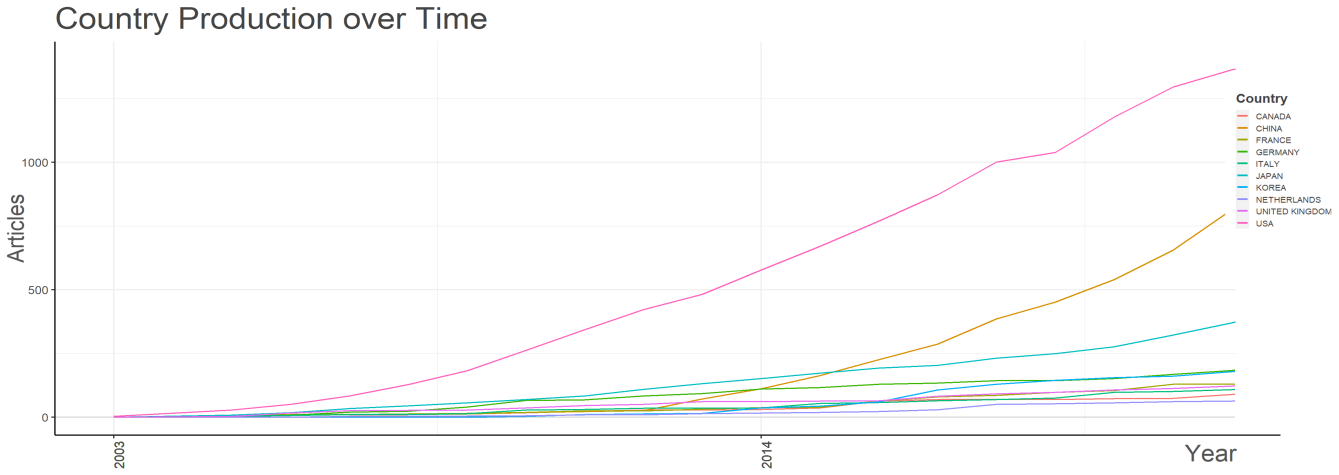


**Supplementary Figure 3.** Country Production over Time：Use Bibliometrix: R-tool version 4.1.4 to visualize the year-by-year growth in publications of the top 10 countries.


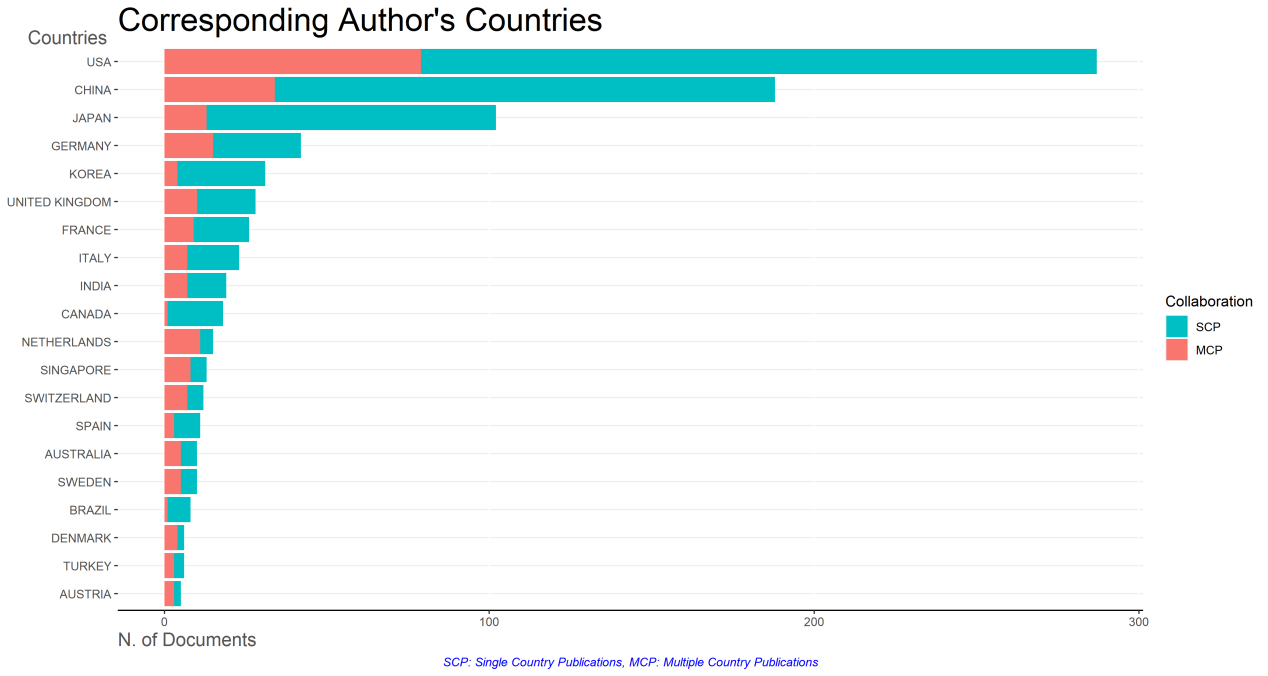


**Supplementary Figure 4.** Corresponding Author’s Countries：Use Bibliometrix: R-tool version 4.1.4 to visualize the top 20 countries with the most published articles

（Scp：Single Country Publications Mcp：Multiple Country Publications）


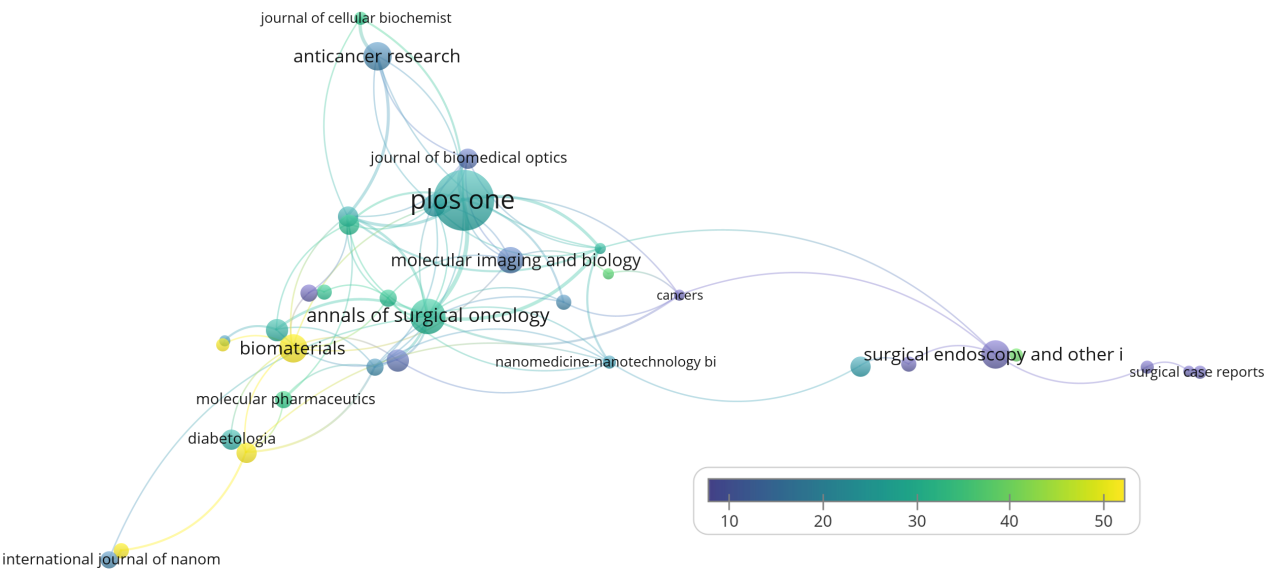


**Supplementary Figure 5.** Journal Collaboration Network Co-occurrence Graph：co-occurrence network visualization of journals with more than 5 published articles using Vosviwer. The larger the node, the greater the number of publications. The color of the node represents the number of citations per article. The darker the color, the fewer the number of citations per article. Thicker lines indicate higher intensity of cooperation.


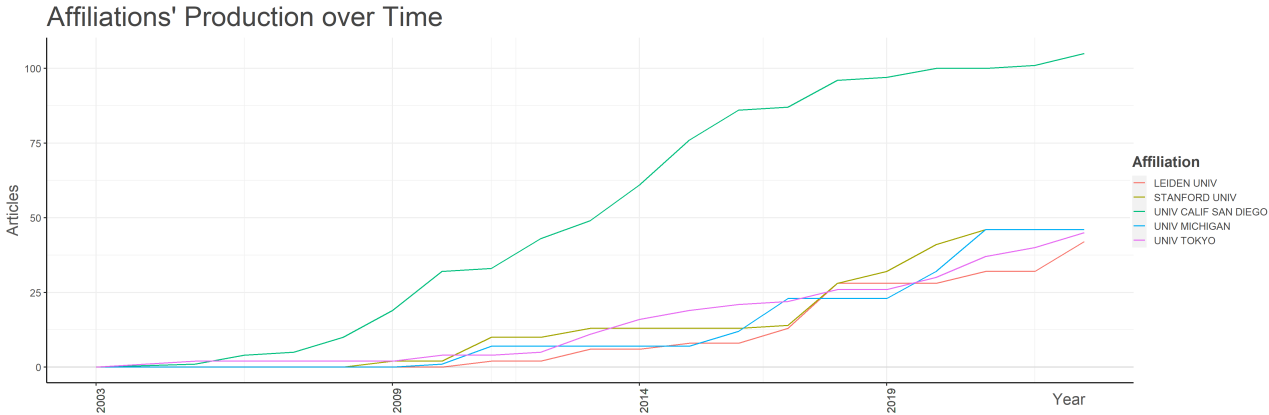


**Supplementary Figure 6.** Affiliations’ Production over Time: Use Bibliometrix: R-tool version 4.1.4 to visualize the year-on-year growth of publications by the top 5 institutions.


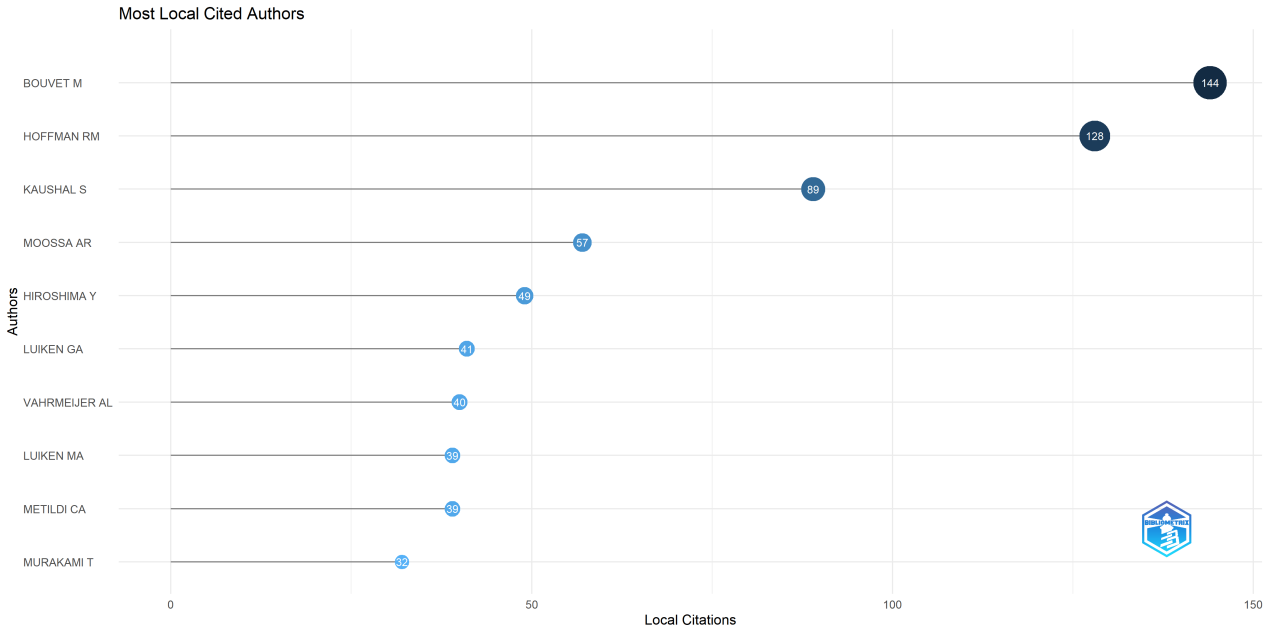


**Supplementary Figure 7.** Most Local Cited Authors:Use Bibliometrix: R-tool version 4.1.4 to visualize the local citations of the TOP10 authors.


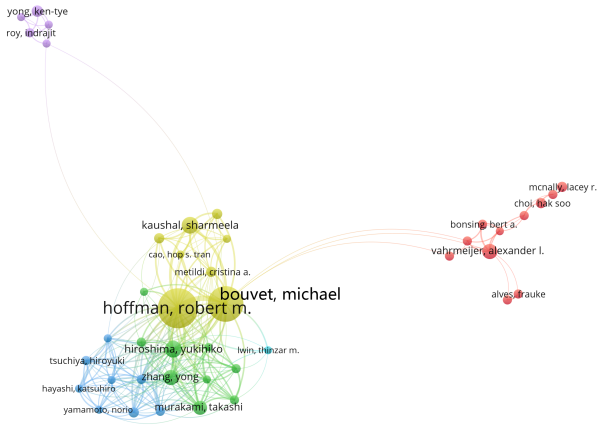

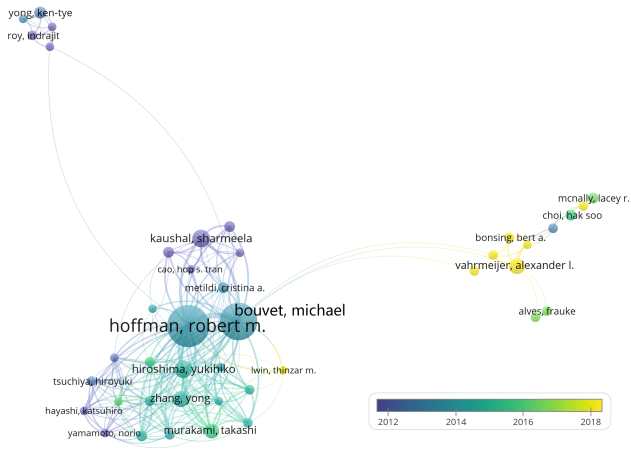


**Supplementary Figure 8.** Author Collaboration Network Co-occurrence Graph:using VOSviewer to visualize the co-occurrence network of authors who have published more than 5 articles. The larger the node, the greater the number of publications. (a) The color represents clustering (b) The color represents the posting time. The earliest node is colored purple, while the latest node is colored yellow.


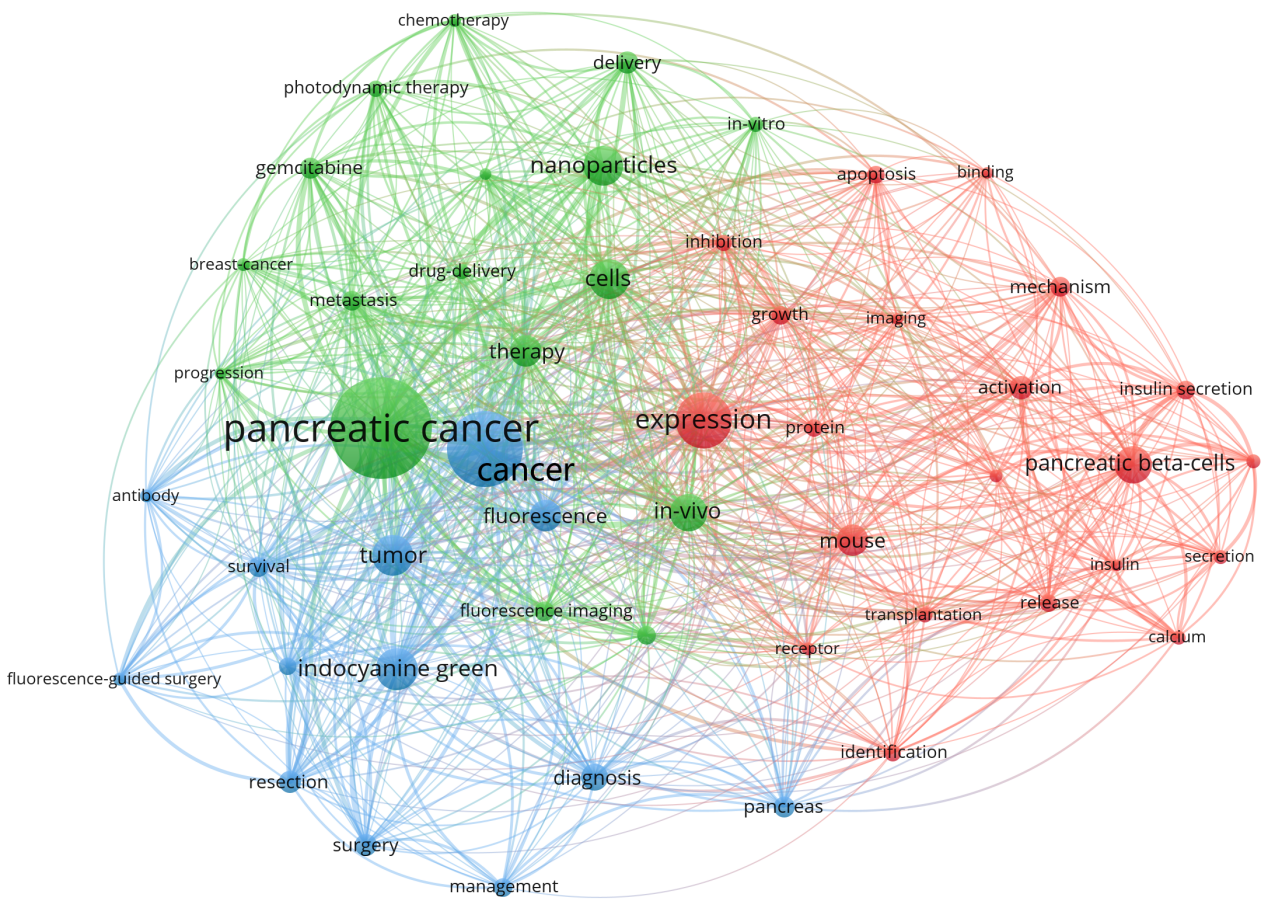


**Supplementary Figure 9.** Keyword Network Co-Occurrence Graph: keywords exceeding 20 occurrences are depicted in co-occurrence networks via VOSviewer, where node size correlates with occurrence frequency, and nodes with the same color belong to the same cluster.


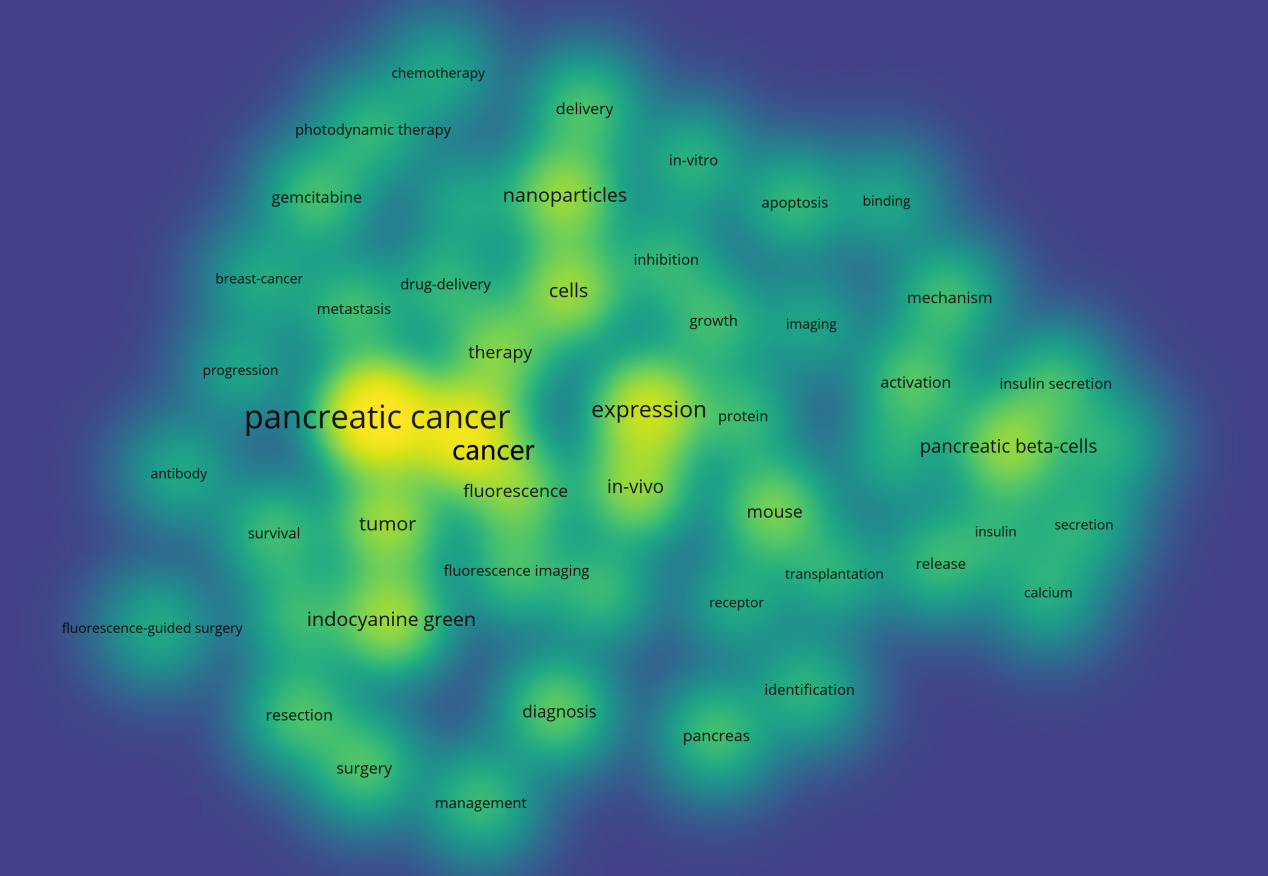


**Supplementary Figure 10.** Keyword co-occurrence density Graph


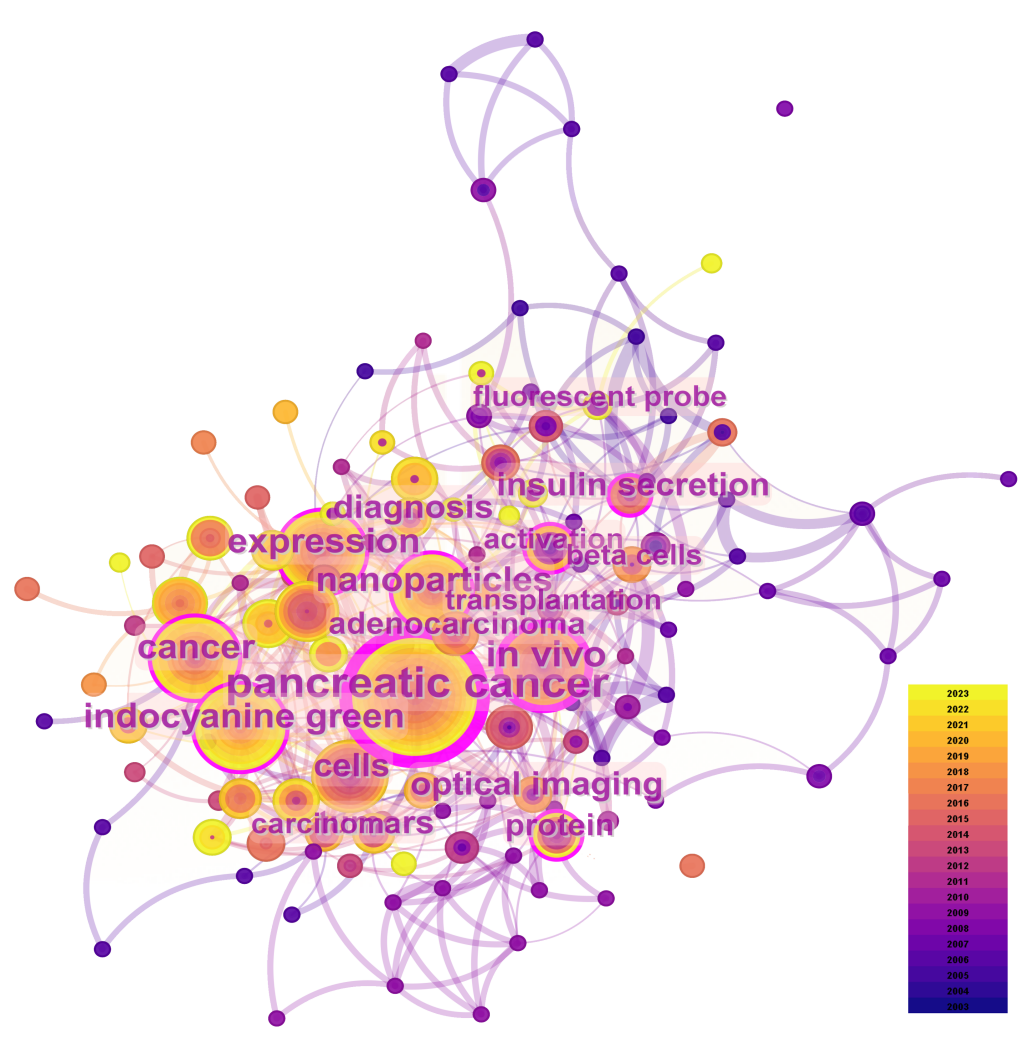


**Supplementary Figure 11.** Keyword Network Co-Occurrence Graph: using CiteSpace to visualize the keywords with TOP10% frequency of occurrence. Each node represents a keyword, the size of the node represents the frequency of occurrence of the keyword, and the annual rings of different widths of the nodes represent occurrences in different years. The purple outer circle marks centrality. The thicker the purple outer circle, the more important the keyword is in the network diagram.
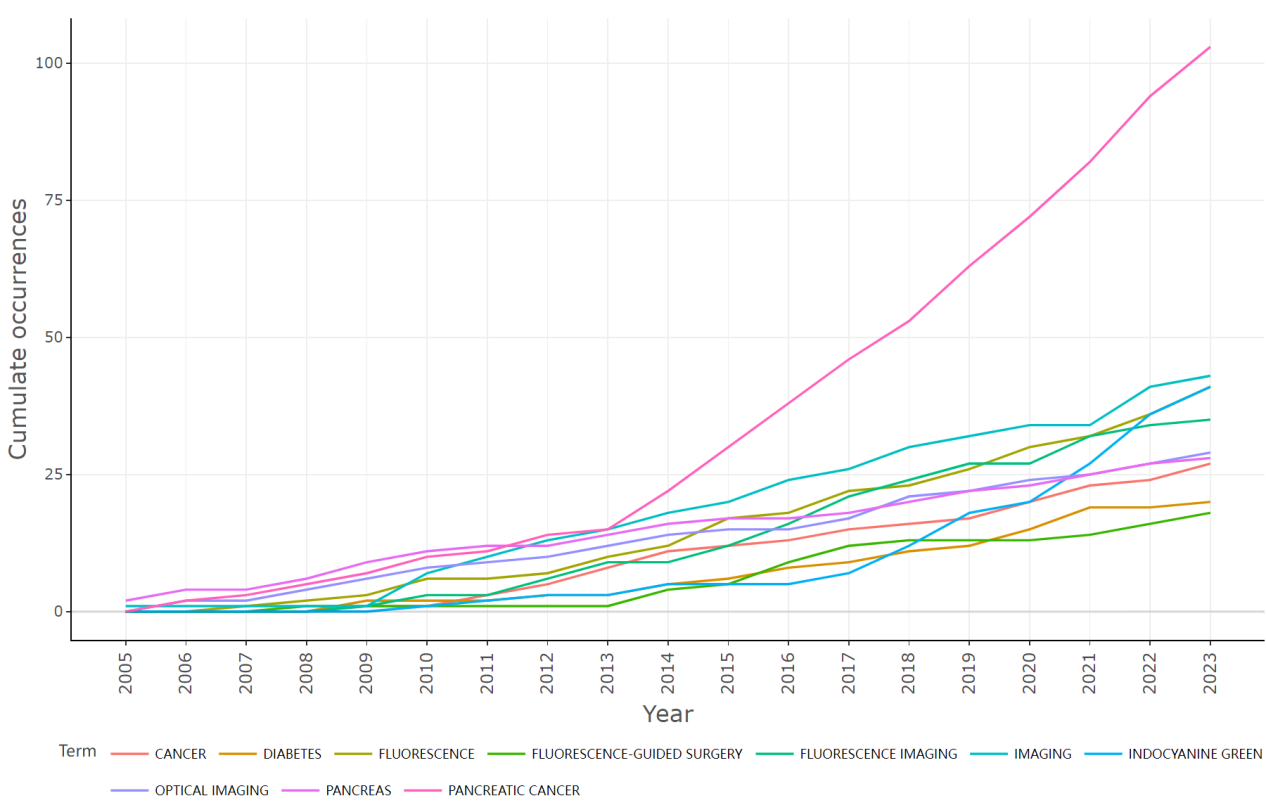


**Supplementary Figure 12.** Use Bibliometrix: R-tool version 4.1.4 to visualize the frequency of occurrence of TOP10 keywords and the growth of published articles year by year.


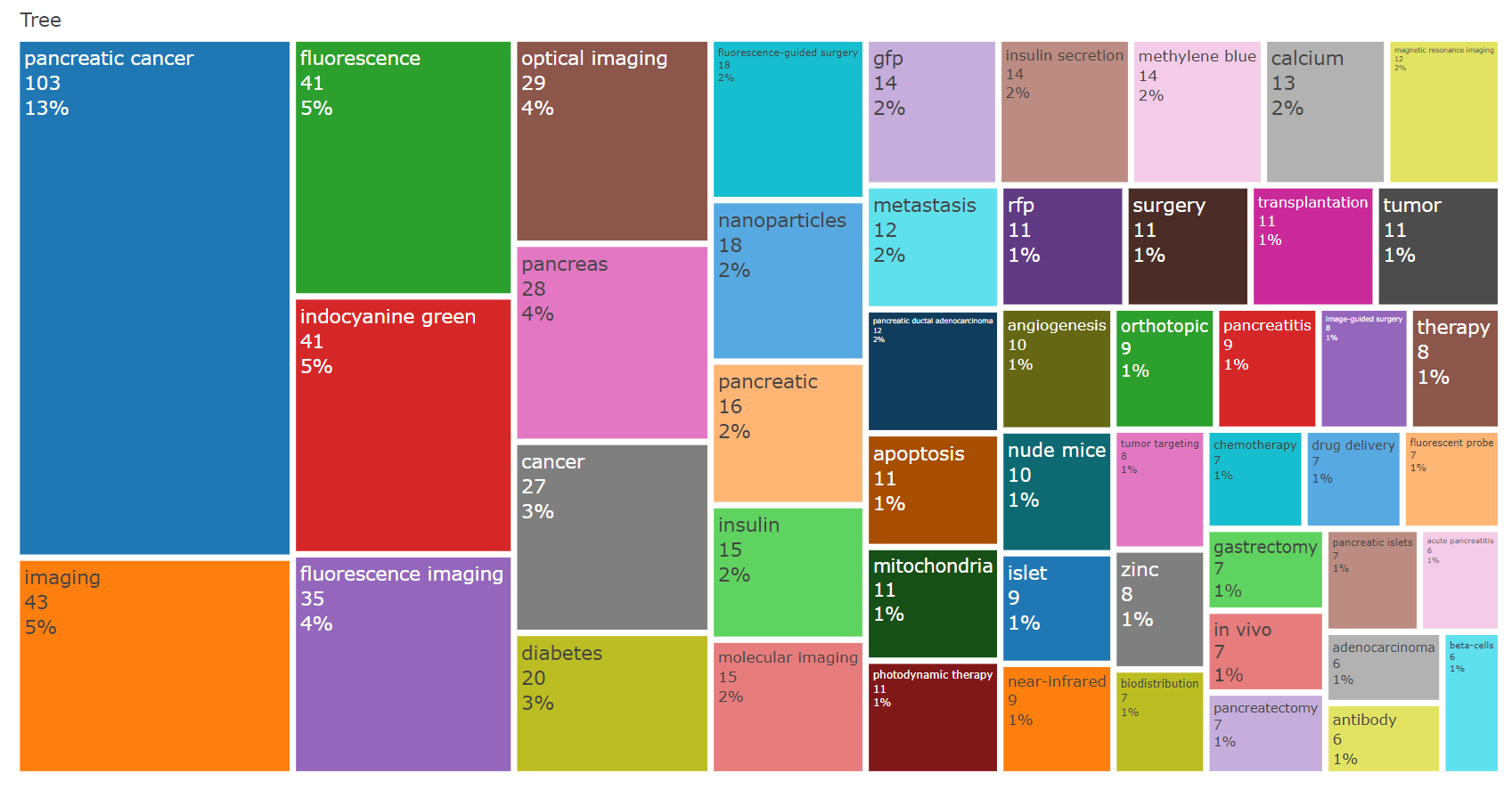


**Supplementary Figure 13.** Visualization of keywords using Bibliometrix: R-tool version 4.1.4


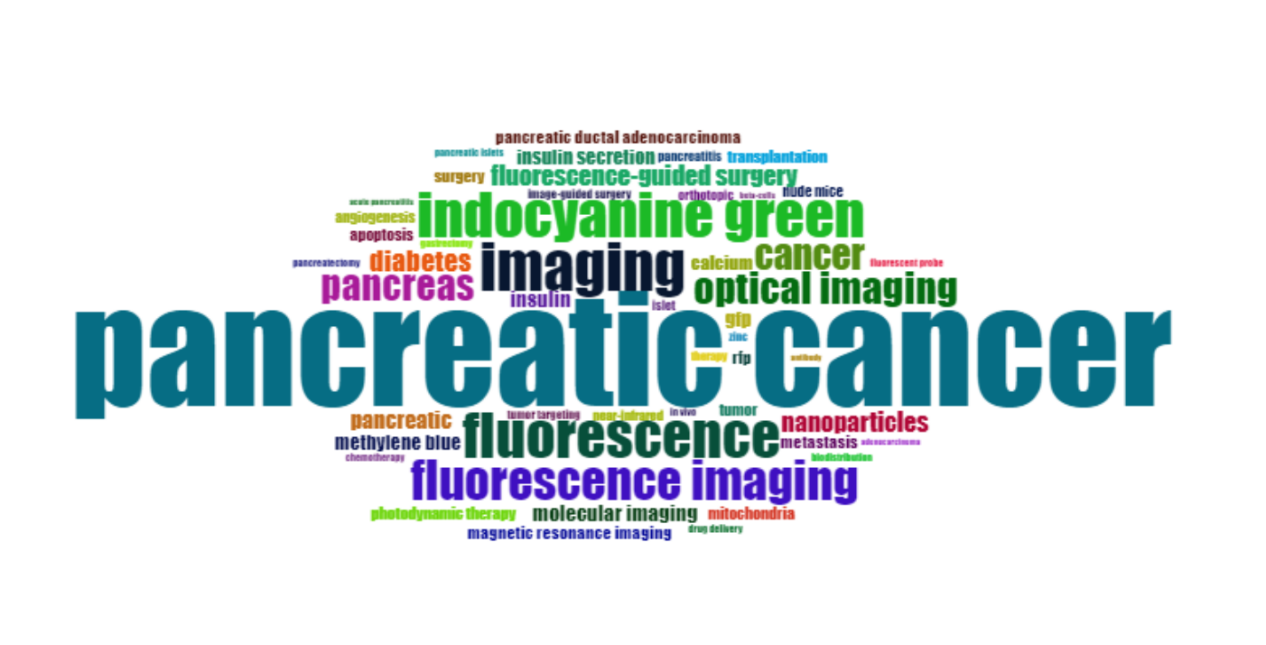


**Supplementary Figure 14.** Visualization of keywords using Bibliometrix: R-tool version 4.1.4


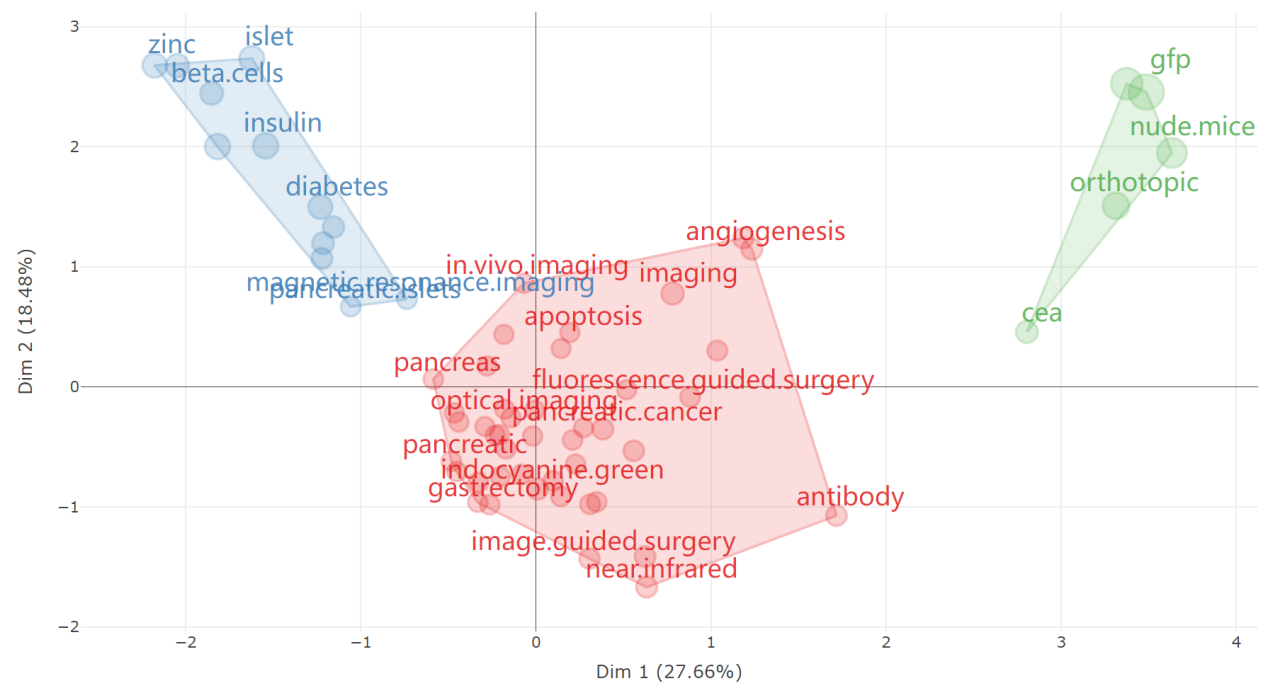


**Supplementary Figure 15.** Keyword clustering graph: Bibliometrix: R-tool version 4.1.4 is used to visualize keyword clustering. Different clusters are represented by different colors.Dim 1 and Dim 2 explained 18.48% and 27.66% of the total variance respectively, with a cumulative explanation rate of 46.14%. The smaller the distance between nodes of different keywords, the more similar they are; conversely, the greater the difference, so the differences between different clusters are more significant.


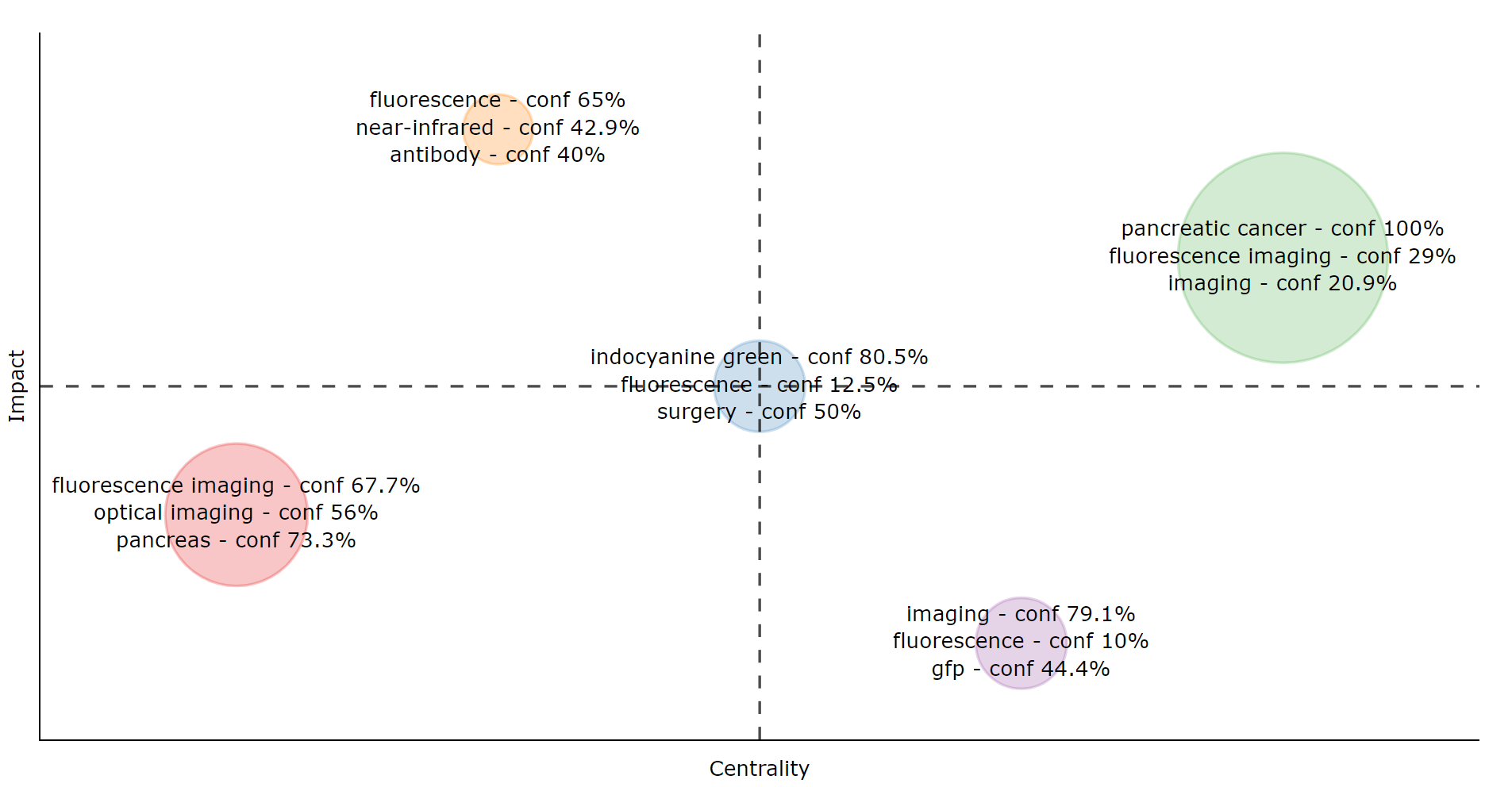


**Supplementary Figure 16.** Bibliometrix: R-tool version 4.1.4 is used to visualize keyword clustering. Different clusters are represented by different colors.


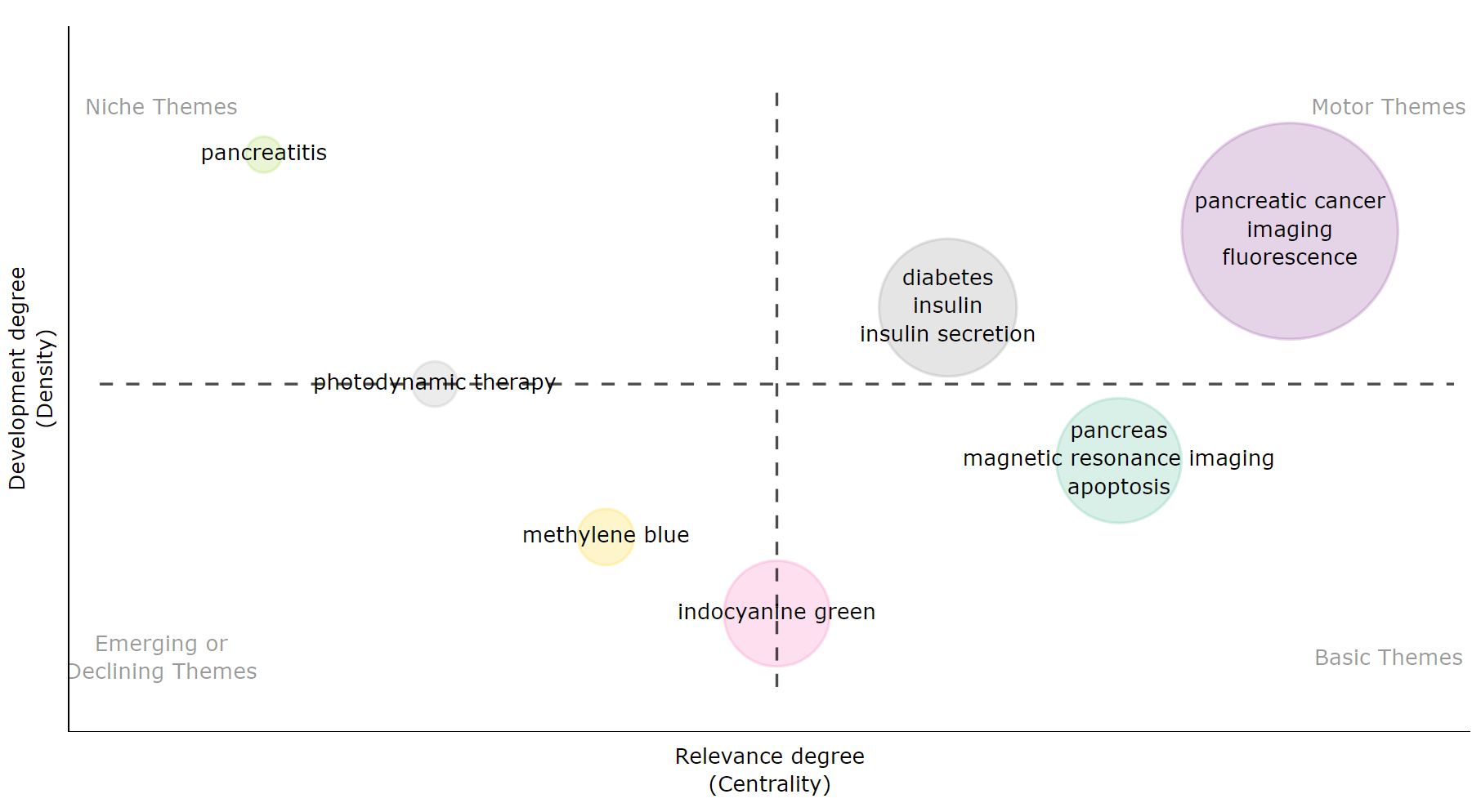


**Supplementary Figure 17.** Thematic map:Using Bibliometrix: R-tool version 4.1.4 to visualize the keyword clustering. The topic in this field has 4 sub-topics, among which pancreatic cancer/imaging/fluorescence and diabetes/insulin/insulin secretion are distributed in the first quadrant and are the core topics in the field.pancreatitis is distributed in the second quadrant, which is an isolated theme in the field; methylene blue is distributed in the third quadrant, which is a new or disappearing theme in the field. Pancreas/magnetic resonance imaging/apoptosis is distributed in the fourth quadrant, which is the basic theme of this field.


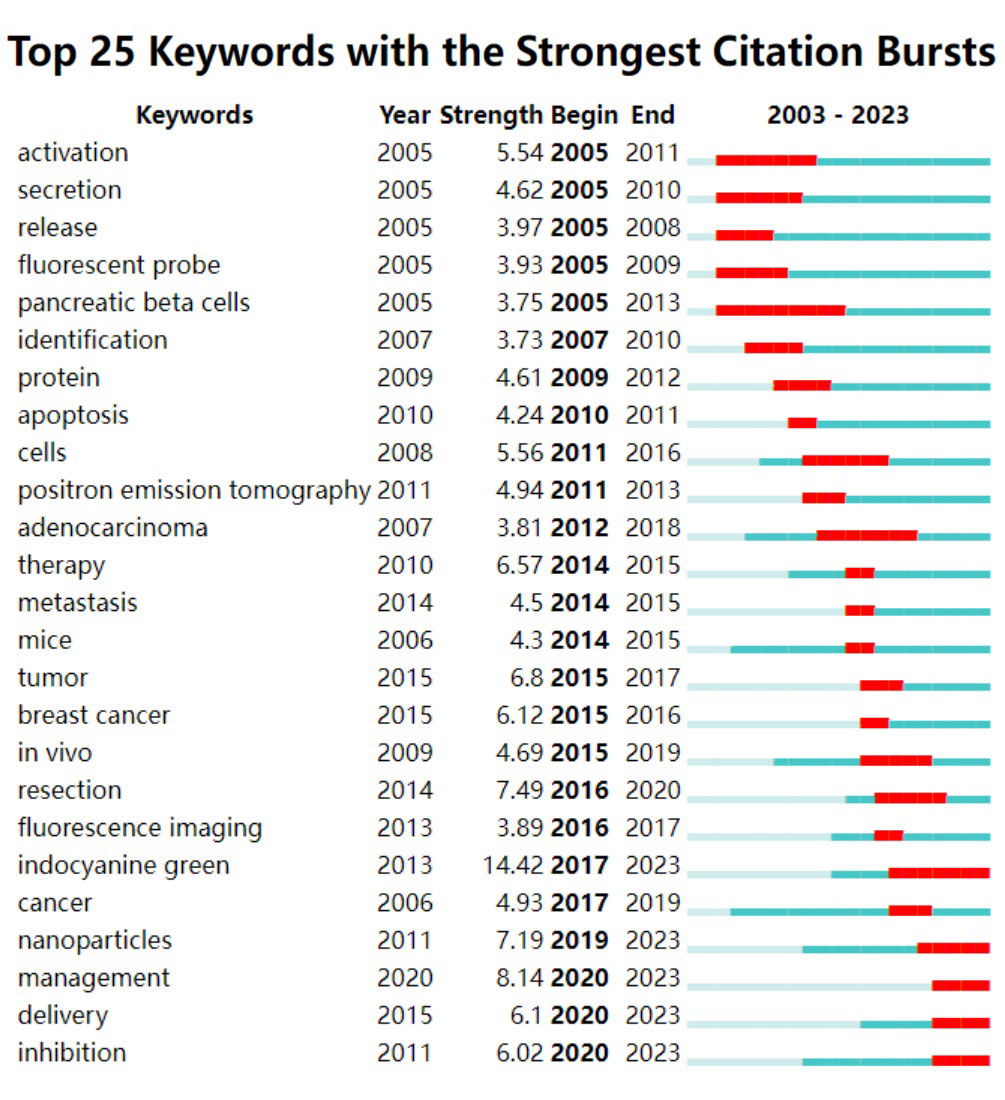
**Supplementary Figure 18.** TOP25 Keywords with the Strongest Citation Bursts: burst word analysis using Citespace. On the right is a timeline view of the emergence of the burst words, with red indicating the time of the keyword burst, blue indicating the time period of existence, and "Strength" indicating the strength of the burst word.
